# Exome Sequencing of Only Seven Qataris Identifies Potentially Deleterious Variants in the Qatari Population

**DOI:** 10.1371/journal.pone.0047614

**Published:** 2012-11-06

**Authors:** Juan L. Rodriguez-Flores, Jennifer Fuller, Neil R. Hackett, Jacqueline Salit, Joel A. Malek, Eman Al-Dous, Lotfi Chouchane, Mahmoud Zirie, Amin Jayoussi, Mai A. Mahmoud, Ronald G. Crystal, Jason G. Mezey

**Affiliations:** 1 Department of Genetic Medicine, Weill Cornell Medical College, New York, New York, United States of America; 2 Department of Genetic Medicine, Weill Cornell Medical College – Qatar, Doha, Qatar; 3 Hamad Medical Corporation, Doha, Qatar; 4 Department of Medicine, Weill Cornell Medical College – Qatar, Doha, Qatar; 5 Department of Medicine, Weill Cornell Medical College, New York, New York, United States of America; 6 Department of Biological Statistics and Computational Biology, Cornell University, Ithaca, New York, United States of America; Institute of Cytology & Genetics SD RAS, Russian Federation

## Abstract

The Qatari population, located at the Arabian migration crossroads of African and Eurasia, is comprised of Bedouin, Persian and African genetic subgroups. By deep exome sequencing of only 7 Qataris, including individuals in each subgroup, we identified 2,750 nonsynonymous SNPs predicted to be deleterious, many of which are linked to human health, or are in genes linked to human health. Many of these SNPs were at significantly elevated deleterious allele frequency in Qataris compared to other populations worldwide. Despite the small sample size, SNP allele frequency was highly correlated with a larger Qatari sample. Together, the data demonstrate that exome sequencing of only a small number of individuals can reveal genetic variations with potential health consequences in understudied populations.

## Introduction

Exome sequencing, in which the coding sequences of the genome are selected from fragmented DNA and analyzed using next-generation sequencing, has led to remarkable insights into the incidence and frequency of polymorphisms within the coding regions of the human genome [1]–[3]. Importantly, exome sequencing has led to the identification of novel genetic variations with potentially deleterious effects on protein structure and function, which are thus of possible importance in health risks [4]. Sampling a diverse global sample of populations by exome sequencing can identify variants that distinguish these populations and can yield insights of medical relevance [5], [6]. However, there are large segments of the world population that have yet to be analyzed by massive parallel sequencing, despite large-scale efforts such as the 1000 Genomes Project (referred to below as 1000 G or 1000 Genomes) [7], the Environmental Genomes Project (http://www.niehs.nih.gov/research/supported/programs/egp/), the Human Genome Diversity Project (http://hagsc.org/hgdp/files.html), and the NHLBI Exome Project (http://www.nhlbi.nih.gov/resources/exome.htm). Identification of specific genetic factors associated with disparate disease risk, incidence, or severity among populations is important for increasing access to genomic medicine [8].

We carried out exome sequencing of 7 randomly chosen Qataris from the 3 Qatari genetic subpopulations [9], representing a region of the Arabian peninsula that is not at present part of any major sequencing effort. The focus was to identify potentially deleterious health-related alleles in Qataris, and to determine whether the prevalence of these alleles is significantly different in Qataris compared to other populations. From these data, potentially deleterious nonsynonymous missense coding polymorphisms were identified. We confirmed that the allelic representation of potentially deleterious missense polymorphisms of health-related interest in this small sample mirrored the frequency of alleles in a larger validation group of Qataris assessed using Affymetrix Genome-Wide SNP Array 5.0 (Affymetrix Inc., Santa Clara, CA) or TaqMan SNP Genotyping Assay (Life Technologies Corp., Carlsbad CA). The representation of SNP alleles in our small sample was compared to the allele frequencies estimated using data from the 1000 Genomes Project (ftp://ftp-trace.ncbi.nih.gov/1000genomes/ftp/release/).

Overall, the data identified numerous potentially deleterious SNPs at significantly elevated frequency in the Qatari population compared to Europeans, Asians, Africans, and recently admixed Americans. These data demonstrate the power of exome sequencing, where sequencing of only a small number of individuals can be used to identify some of the potentially deleterious health-related alleles in a population and to identify allelic variation that differentiate these peoples from African, European, Asian, and American populations. The results provide insight into this population that, despite its importance in the story of human genetic variation [10], [11], has not been represented in the major genome consortium sequencing efforts [5]–[7].

## Results

### Classification of the Potentially Deleterious SNPs Identified in Seven Qatari Exomes

**Figure 1:**
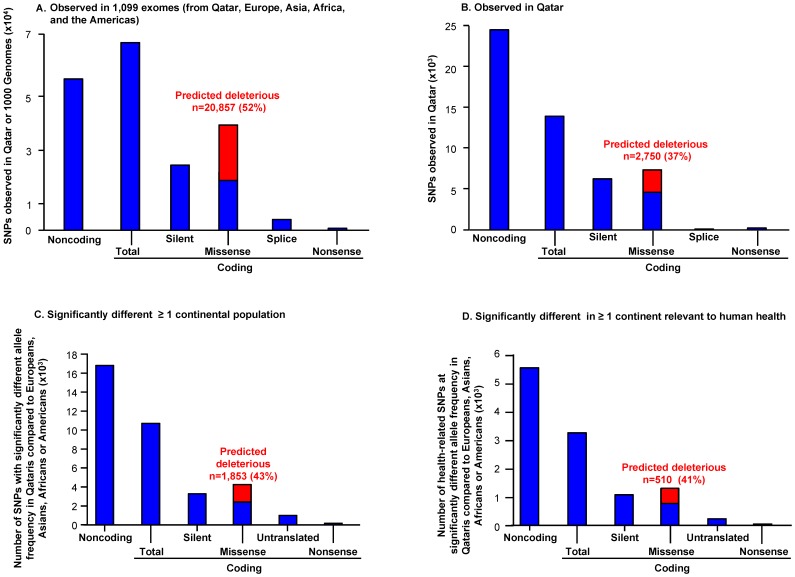
Functional classification of single nucleotide polymorphism (SNP) sites in seven Qatari exomes. Genotypes for 126,924 SNPs in target exons ±500 bp were confidently called as ref/ref, ref/alt or alt/alt (where ref = GRCh37 reference allele and alt = non-reference allele) using GATK [33] and classified using databases of SNP function (NCBI dbSNP build 134, SIFT online webserver [34], and GATK VariantAnnotator function [33]. Shown are bar plots of: **A.** SNPs observed in ≥1 of 1,099 exomes [QE7 and 1000 G]; **B.** SNPs identified in ≥1 of 14 QE7 alleles; **C.** SNPs significantly higher or lower in QE7 *vs* at least one population; and **D.** Subset of significantly higher or lower SNPs in genes with a health-related role (OMIM [12], HGMD [37], PharmGKB [38] or HUGE [39]). In the four plots, the x-axis lists the functional categories (noncoding, coding, silent, missense, splice, nonsense) and the y-axis the number of SNPs. There were 20,857 (52%) missense SNPs predicted deleterious by SIFT [34] or PolyPhen2 [35] polymorphic in 1,099 exomes (QE7 and 1000 G), a subset 2,750 polymorphic in QE7 with ≥1 of 14 alleles (Table 1). There were 1,853 significantly higher or lower missense SNPs predicted deleterious by SIFT [34] or PolyPhen2 [35] polymorphic in 1,099 exomes (QE7+1000 G), and a subset of 510 relevant to health; see Table 2). Red = predicted deleterious SNPs.

Of the 126,924 sites polymorphic in the seven analyzed Qatari exomes (QE7) or that were fixed and present in 1000 Genomes, 20,857 were missense nonsynonymous SNPs predicted to be potentially deleterious by SIFT or PolyPhen2 (633 novel and 20,224 in dbSNP 134, Figure 1A). Of these potentially deleterious SNPs, 2,750 (13% of 20,857) were observed in ≥1 of the 14 QE7 alleles (Figure 1B), and 339 were observed in ≥6 of the 14 QE7 alleles (1.6% of 20,857; Table 1). These SNPs were subclassified using databases of disease and metabolism as: (1) not previously associated with nor within a health related gene (1,969, 72% in ≥1 of the 14 QE7 alleles; 240 (9%) in ≥6 of QE7); (2) in a gene previously linked to human health, but a different SNP than previously reported (650, 24% in ≥1/14; 74 (3%) in ≥6/14 alleles); and (3) the gene and SNP have previously been linked to human health (131, 4% in ≥1/14; 25 (1%) in ≥6/14 alleles; Table 1). A subset of these SNPs were validated by Affymetrix Genome-Wide SNP Array 5.0 (Affymetrix Inc., Santa Clara, CA) genotyping in n = 149 Qatari (QA149) and by TaqMan genotyping in n = 86 Qatari.

**Table 1:** Potentially Deleterious Missense Coding SNPs in the Qatari Genome Identified by Exome Sequencing^1^.

Functional classification^2^	Potentially deleterious alternate allele observed in QE7 n (%)^3^	
	≥1 of 14	≥6 of 14
Total potentially deleterious missense coding SNPs	2,750 (100%)	339 (100%)
Not previously associated with nor within a health-linked gene	1,969 (72%)	240 (71%)
In a gene previously linked to human health, but a different SNP than previously reported	650 (24%)	74 (22%)
The gene and SNP have been previously linked to human health	131 (5%)	25 (7%)

1 In order to identify potentially deleterious missense health-linked SNPs in Qatar, genotypes of the 2,750 predicted to be potentially deleterious alternate alleles observed in QE7 were subdivided by frequency [≥1/14 or ≥6/14 alternate allele frequency] and by functional category.

2 In order to identify potentially deleterious SNPs of medical interest in Qatar, the 2,750 predicted to be potentially deleterious SNPs were subclassified into 3 groups based on prior link of the gene or SNP to a health-related phenotype using four major databases of disease and metabolism SNPs (OMIM [12], HGMD [37], PharmGKB [38] and HUGE [39]). 1^st^ row - total number of potentially deleterious SNPs; 2^nd^ row - number of potentially deleterious of SNPs where no SNP in the gene has been previously associated with a phenotype relevant to human health; 3^rd^ row - SNPs in genes linked to human health, but the SNP has not been previously tested for phenotypic effect; and 4^th^ - number of potentially deleterious SNPs where the specific SNP and gene has been reported to be health-linked. SNPs in the fourth row (previously identified) are not counted in the third row (in a gene, but not SNP, previously linked).

3 1^st^ column - the 2,750 SNPs where the potentially deleterious alternate allele was observed at least once in QE7 (≥1 of 14), representing 2% of the sites confidently genotyped in QE7, subdivided by the health-linked classification described above. 2^nd^ column - the 339 potentially deleterious alleles observed at least 6 times in QE7 (12% of the 2,750), subdivided by the health-linked classification described above. For each column, percentages are based on the total in the first row for that column.

#### Potentially deleterious SNPs where the gene and SNP have been previously linked to human health

**Figure 2:**
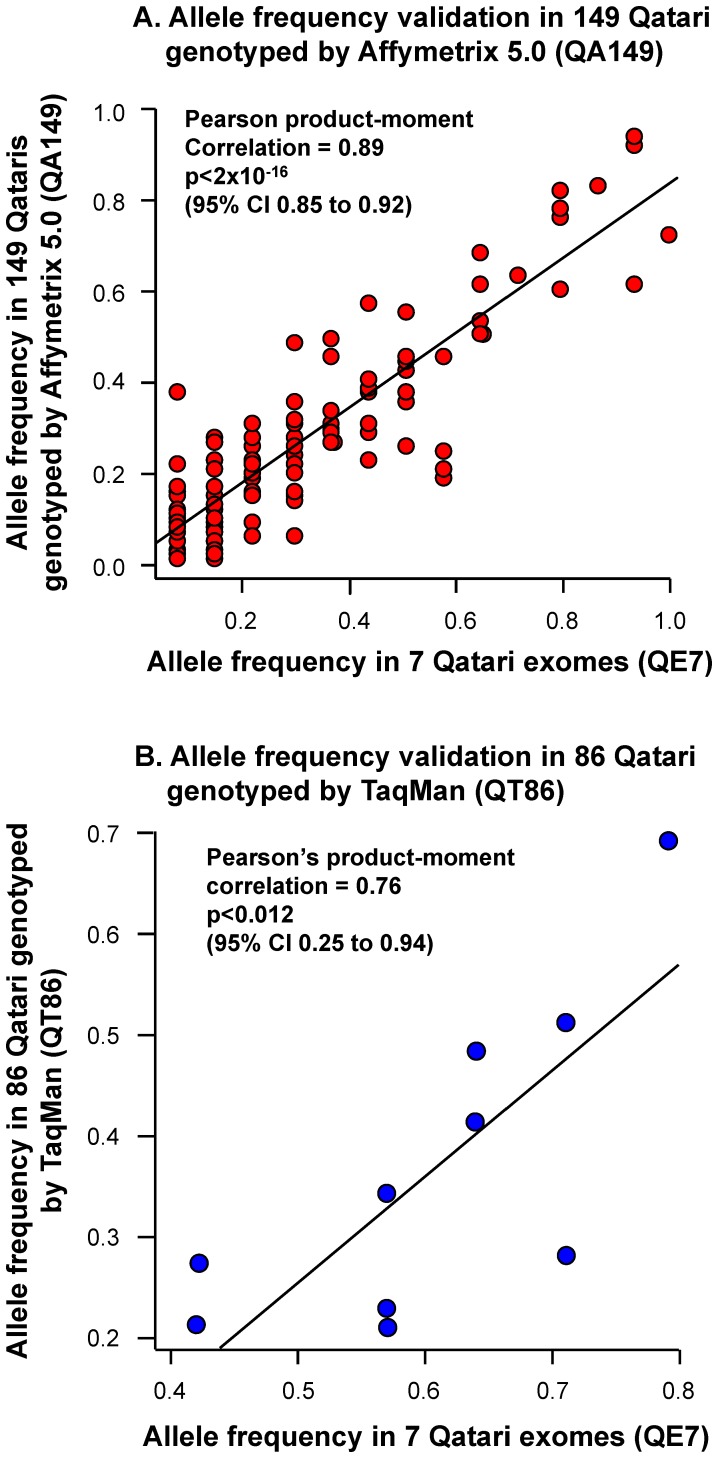
Validation of allele frequency for potentially deleterious nonsynonymous missense SNPs observed in n = 7 Qatari exomes using Affymetrix 5.0 array genotyping of n = 149 Qataris or TaqMan genotyping of n = 86 Qataris (n = 82 overlapping). A. To confirm the allele frequency estimates for the Qatari population based on the number of alleles observed in QE7 (n = 14 alleles) for potentially deleterious SNPs, the QE7 allele frequency observed in at least 1 of 14 (7%) QE7 alleles was compared to the allele frequency in QA149 (n = 298 alleles) generated using Affymetrix 5.0 SNP microarrays. Of the 2,750 potentially deleterious nonsynonymous SNPs identified in QE7, 149 probes were on the Affymetrix 5.0 array. Shown is the QE7 allele frequency along the x-axis and the QA149 allele frequency along the y-axis for 131 SNPs, excluding 18 Affymetrix 5.0 SNPs where the QE7 allele frequency could not be validated due to partial missing genotypes. B. Validation of allele frequency for potentially deleterious nonsynonymous missense SNPs significantly higher or lower in Qatari exomes using TaqMan genotyping of n = 86 Qataris. To confirm the allele frequency estimates for the Qatari population based on the number of alleles observed in QE7 (n = 14 alleles) for deleterious SNPs in Table 4, the QE7 allele frequency observed in at least 1 of 14 (7%) QE7 alleles was compared to the allele frequency in QT86 (n = 172 alleles) generated using TaqMan. Shown is the QE7 allele frequency along the x-axis and the QT86 allele frequency along the y-axis.

Of the 650 potentially deleterious missense coding SNPs identified in ≥1 of 14 QE7 alleles, 131 were on the Affymetrix 5.0 microarray, and thus could be confirmed in the QA149 Qatari validation set (Table 1, Figure 2A). Of these 131 potentially deleterious SNPs, 49 had been previously linked to human health. Of these, we selected 10 of potential medical relevance in Qatar to highlight (Table 2; see Web Resource S1 for all potentially deleterious SNPs). Among these 10 SNPs, several are relevant to disorders common in Qataris, including type 2 diabetes (PPARG, PPP1R3A), cardiovascular disease (PON2, MTR), hypertension (ULK4), pulmonary disease (CDC6) and neurologic disease (BDNF).

**Table 2:** Affymetrix Microarray Validation of Qatari Exome Potentially Deleterious SNPs Where the SNP and Gene Have Been Previously Linked to Human Health^1^.

									Allele frequency		
Gene^2^		SNP^3^							QE7 exome sequencing (n = 14 alleles)^6^	QA149 Affymetrix genotyping (n = 298 alleles)^7^	
Symbol	Name	Amino acid substitution	dbSNP rsID	Chromosome	Position	Ref> alt	Risk allele	Health-associated phenotype^4,5^	Alt	Alt	Risk
PPARG	Peroxisome proliferator-activated receptor gamma	Pro12Ala	rs1801282	3	12393125	C>G	C	Type 2 diabetes	0.07	0.05	0.95
PON2	Paraoxonase 2	Ser311Cys	rs7493	7	95034775	G>C	G	Coronary artery disease	0.29	0.28	0.72
NAT2	N-acetyltransferase	Arg197Gln	rs1799930	8	18258103	G>A	A	Slow metabolism of clonazepam	0.14	0.23	0.23
MTR	5-methyltetrahydrofolate-homocysteine methyltransferase	Asp473Gly	rs1805087	1	237048500	A>G	G	Cardiovascular disease	0.29	0.22	0.22
NQO1	NAD(P)H dehydrogenase, quinone 1	Pro187Ser	rs1800566	16	69745145	G>A	A	Increased risk of benzene poisoning, colorectal cancer, poor survival in breast cancer	0.14	0.21	0.21
ULK4	Unc-51-like kinase 4	Lys569Arg	rs3774372	3	41877414	T>C	C	Diastolic blood pressure	0.57	0.19	0.19
CDC6	Cell division cycle 6	Val441Ile	rs13706	17	38457151	G>A	A	Rate of decline in ex-smokers with COPD	0.29	0.14	0.14
PARP1	Poly (ADP-ribose) polymerase 1	Val762Ala	rs1136410	1	226555302	A>G	G	Increased risk of cancer in Asians, decreased risk of cancer in Europeans	0.14	0.13	0.13
BDNF	Brain-derived neurotrophicfactor	Val66Met	rs6265	11	27679916	C>T	T	Anxiety, depression disorders, motor function	0.07	0.11	0.11
PPP1R3A	Protein phosphatase 1,regulatory subunit 3A	Asp905Tyr	rs1799999	7	113518434	C>A	A	Type 2 diabetes, insulin resistance	0.14	0.10	0.10

1 Analysis of the exomes in the QE7 14 alleles identified 131 missense coding SNPs where the SNP and gene have been previously identified as linked to human health (Table 1, 4^th^ row). To validate this observation in a larger group of Qataris, the Affymetrix Genome-Wide SNP Array 5.0 was used to assess an independent group of 149 Qataris (QA149, 298 alleles). Of the 2,750 missense potentially deleterious SNPs identified in at least 1 of the QE7 14 alleles, 131 were on the microarray. Of these, 49 were in genes linked to human health, including 16 where both the gene and the SNP are linked to human health. Of these 16, listed are 10 chosen as examples of missense SNPs linked to human health.

2 Gene symbol and name obtained from the Consensus Coding Sequence (CCDS) NCBI database [32], amino acid substitution position and residues obtained from dbSNP when available, otherwise SIFT online webserver [34]. Transcript position and amino acid substitution were verified to be consistent with the literature.

3 SNP information includes chromosome amino acid substitution, dbSNP build 134 rsID if available, chromosome, position in GRCh37 human reference genome assembly, reference and alternate allele in QE7. Ref = references; alt = alternative.

4 Phenotype information from OMIM [12], HGMD [37], PharmGKB [38] or HUGE [39] database.

5 For more details and references, see Details S1.

6 Shown is the alternate allele frequency determined by exome sequencing in QE7 individuals.

7 Shown is the risk allele frequency in the validation set of QA149 individuals (n = 149 Qatari, 298 alleles). Failed genotypes are accounted for in the allele frequency. For statistical comparisons of the QE7 and QA149 allele frequencies, see Figure 2.

#### Potentially deleterious SNPs when the gene but not the SNP has been previously linked to human health

Of the 650 potentially deleterious missense coding SNPs identified in ≥1 of 14 QE7 alleles where the SNP was in a gene previously linked to human health, but the potentially deleterious SNP was different than previously reported, 33 were on the Affymetrix 5.0 microarray and could be validated in the QA149 Qataris (Web Resource S1). Of these 33, the genes containing 13 were described in the OMIM database of Mendelian Disorders [12]. Of these SNPs, 10 of potential medical relevance in Qatar were further assessed (Table 3). Two of these genes have been related to ophthalmologic disorders, including age related macular degeneration (HMCN1) and keratoconus (VSX1) and several have been related to neurologic disorders (IKBKAP, SGCG, SACS, ARHGEF10, and CACNA1S).

**Table 3:** Affymetrix Microarray Validation of Qatari Exome Predicted Deleterious SNPs in Genes Linked to Human Health, but a Different SNP than Previously Reported^1^.

											Allele frequency	
Gene^2^		SNP^3^									QE7 exome sequencing (n = 14 alleles)^6^	QA149 Affymetrix genotyping (n = 298 alleles)^7^
Symbol	Name	Amino acid substitution	dbSNP rsID	Chromosome	Position		Ref> alt		Health-associated phenotype^4,5^		Alt	Alt
HMCN1	Hemicentin	Gln4437ARG	rs10911825	1	186101539	A>G		Age related macular degeneration		0.50		0.43
IKBKAP	Inhibitor of kappa light polypeptidegene enhancer in B cells, kinasecomplex-associated protein	Cys1072Ser	rs3204145	9	111651620	A>T		Familial dysautonomia		0.14		0.27
VSX1	Visual system homeobox gene 1	Arg217His	rs6138482	20	25059442	C>T		Keratoconus, polymorphous corneal dystrophy		0.21		0.19
EVC	Ellis-van creveld syndrome	Gln74Pro	rs2291157	4	5721021	A>C		Ellis-van Creveld syndrome; Weyers acrodental dysostosis		0.07		0.17
SGCG	Sarcoglycan, gamma	Arg116His	rs17314986	13	23824818	G>A		Muscular dystrophy, limb-girdle, type 2c		0.07		0.16
SACS	Sacsin	Asn232Lys	rs2031640	13	23930055	A>T		Spastic ataxia, Charlevoix-Saguenay type		0.07		0.11
OSMR	Oncostatin m-specific receptor	Glu527Lys	rs10941412	5	38919158	G>A		Primary cutaneous amyloidosis		0.14		0.10
ARHGEF10	Rho guanine nucleotide exchange factor 10	Ser980Ala	rs17683288	8	1877480	T>G		Slow nerve conduction velocity, autosomal dominant		0.14		0.09
CACNA1S	Calcium channel, voltage-dependent, l type, alpha-1s	Arg1539Cys	rs3850625	1	201016296	G>A		Hypokalemic periodic paralysis, thyrotoxic periodic paralysis, malignant hyperthermia		0.07		0.08
RSPH4A	Radial spoke head 4, Chlamydomonas, homolog A	Asn627His	rs9488991	6	116951678	A>C		Ciliary dyskinesia, primary		0.07		0.05

1 Analysis of the exomes in the QE7 14 alleles identified 650 missense coding SNPs where the gene has been previously identified as linked to human health, but the missense SNP is different than that previously reported (Table 1, row 3). To validate this observation in a larger group of Qataris, the Affymetrix Genome-Wide SNP array 5.0 was used to assess an independent group of 149 Qataris (QA149, 298 alleles). Of the 2,750 missense potentially deleterious SNPs identified in at least 1 of the QE7 14 alleles, 131 were on the microarray. Of these, 49 were in genes linked to human health, including 33 where the gene is linked to human health, but the reported link was for a different SNP. Of these 33, listed are 10 chosen as examples of missense SNPs linked to human health that are extensively documented in the OMIM database (3).

2 Gene symbol and name obtained from the Consensus Coding Sequence (CCDS) NCBI database [32], amino acid substitution position and residues obtained from dbSNP when available; otherwise SIFT online webserver [34]. Transcript position and amino acid substitution was verified to be consistent with the literature.

3 SNP information includes amino acid substitution, dbSNP build 134 rsID if available, chromosome, position in GRCh37 human reference genome assembly, reference and alternate allele in QE7. Ref = references; alt = alternative.

4 Phenotype information from OMIM [12], HGMD [37], PharmGKB [38] or HUGE [39] database.

5 See Details S1.

6 Shown is the alternate allele frequency determined by exome sequencing in QE7 individuals.

7 Shown is the alternate allele frequency in the validation set of QA149 individuals (n = 149 Qatari, 298 alleles). Failed genotypes are accounted for in the allele frequency. For statistical comparisons of the QE7 and QA149 allele frequencies, see Figure 2.

### Correlation between QE7 and Larger Qatari Sample Allele Frequency

**Figure 3:**
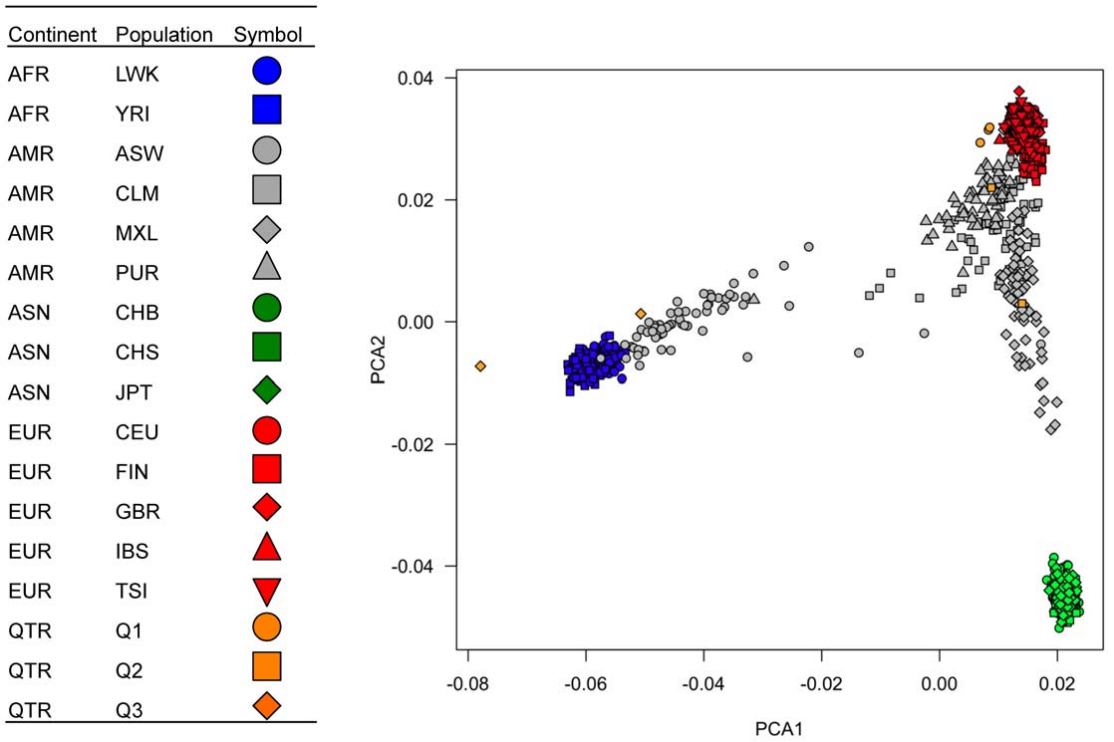
Principal component analysis (PCA) validation of exome genotypes for the QE7 individuals. In order to verify the overall quality of the genotyping call set, the seven Qatari exomes were compared to 1,092 individuals from four continents (1000 Genomes Project October 2011 Integrated Phase 1 Variant Set Release) at 18,865 SNPs segregating in both QE7 and 1000 Genomes that are present in dbSNP build 134 using SMARTPCA [14]. Plotted is PCA 1 (x-axis) *vs* PCA 2 (y-axis). Individuals are color-coded by continent of origin (European = red, Asian = green, African = blue, American = grey, Qatar = orange). Clustering of the Qatari individuals was verified to be consistent with our prior report [9], where Q1 cluster near Europeans, Q2 in between Q1 and Asians, and Q3 between Q1 and Africans.

One of the important observations in the present study is the remarkable correlation of the allele frequency of potentially deleterious nonsynonymous SNPs described by sequencing only 7 Qatari exomes with a larger validation population of Qataris (Figure 2). While exome sequencing of only 7 individuals in a population only provides a snapshot of some of the potentially deleterious SNPs in the population, we asked the question: would sequencing of only 7 subjects in each of the continental populations also provide a valid snapshot of at least some of the potentially deleterious SNPs in these populations? To answer this question, using 1000 Genomes Project genotypes at 20,381 potentially deleterious SNPs genotyped in the QE7, we calculated the allele frequency correlation between a sample size of 7 *vs* a larger Qatari sample of n = 48, n = 174 or n = 193, depending on the number of available samples, using Pearson’s product-moment method (Table S4). For comparison, we also compared the allele frequency correlation for seven individuals selected from 1000 Genomes populations, continents, and “World mix 1 and 2” of 3 European, 2 Asian and 2 African individuals with a larger sample of 193 from a random selection of the larger populations. These mixes of 3 European, 2 Asian, and 2 African were similar to a mix of 3 Q1, 2 Q2 and 2 Q3 based on the observation that SNPs used to classify Qatari into ancestry groups are effective at classifying 1000 Genomes individuals into European, Asian, and African clusters using both STRUCTURE [13] quantification of genome-wide admixture proportions (Figure S2) and SMARTPCA [14] principal component analysis (Figure 3).

**Figure 4:**
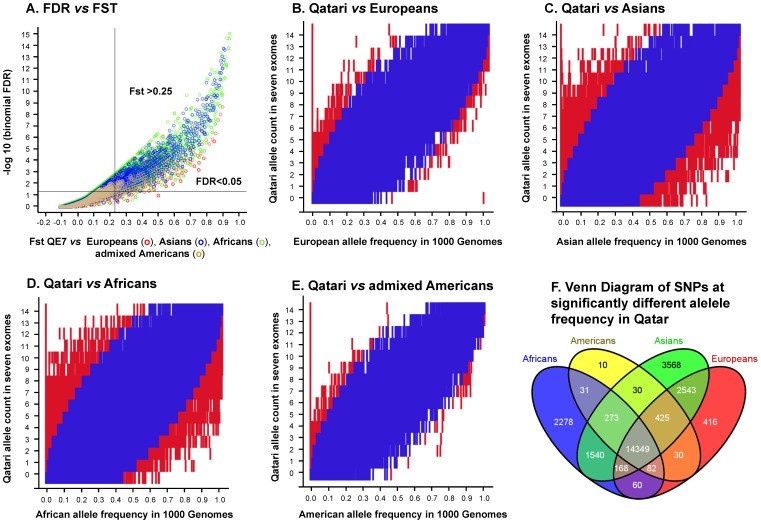
Identification of autosomal exome SNPs in the QE7 Qatari individuals with an allele frequency distinct from at least one continent (Europe, Asia, Africa, the Americas) as estimated from exomes *vs* 1000 Genomes. **A.** Illustration of threshold selection. Fixation index (Fst; x-axis) and -log10 (q-values; y-axis) for a binomial test for all SNPs assessed versus each continent (red = QE7 *vs* Europeans, green = QE7 *vs* Asians, blue = QE7 *vs* Africans, tan = blue = QE7 *vs* Americans). Shown is the threshold selected for identifying enriched SNPs; Fst >0.25 [43] and FDR <0.05 [44]. **B–E.** Heat maps of the false discovery rate [44] for enrichment of higher or lower than expected number of alternative alleles tested on 126,924 exome SNPs. Shown is the allele counts for the 7 Qatari exomes (y axis) *vs* the continental alternative allele frequency in 1000 Genomes continental populations (x axis). The map shows combined FDR and Fst thresholds for all SNPs (enriched = red = Fst >0.25 and FDR <0.05; not enriched = blue = Fst <0.25 or FDR >0.05; white = no observations). **B.** Qataris *vs* Europeans (EUR), **C.** Qataris *vs* Asians (ASN), and **D.** Qataris *vs* Africans (AFR). **D.** Qataris *vs* Americans (AMR). **E.** Venn diagram of 25,803 SNPs enriched in Qataris *vs* at least one continent.

The “World mix 1 and 2″ had the lowest allele frequency correlation observed out of all groups tested (0.85 and 0.86). The highest allele frequency correlation between small and larger samples was for Asians (0.91 and 0.92) and Europeans (0.92), for each population, each continent. The allele frequency correlation for European and Asian populations was, in general, higher than that of African and American populations. By comparison, the Qatari allele frequency correlation was 0.89, higher than a “World mix 2″ but lower than a European or Asian population. The observed correlation was similar for 131 potentially deleterious SNPs validated by Affymetrix 5.0 genotyping in 149 Qataris (Pearson correlation of 0.89; p<2×10^−16^, 95% confidence interval 0.85 to 0.92, Figure 4A) and for 10 potentially deleterious SNPs validated by TaqMan PCR in 86 Qataris (Pearson’s correlation 0.76, p<0.012, 95% confidence interval 0.25 to 0.94; Figure 4B).

### Comparison of the Prevalence of QE7 SNPs to Prevalence of These SNPs in 1000 Genomes

SNPs at significantly different allele frequency that make Qataris distinct from Europeans, Asians or Africans, were identified with an Fst threshold of population differentiation of Fst>0.25 and a Storey q-value multiple testing corrected p value of q<0.05 for the probability of observing *x* alternate alleles in 14 trials, using the 1000 Genomes continental allele frequency as a probability of success (Figure 3). Out of 25,803 SNPs at significantly different allele frequency in Qatar, 77% (19,940) had higher alternate allele frequency in QE7 in the four comparisons (QE7 *vs* Europeans, QE7 *vs* Asians, QE7 *vs* Africans, QE7 *vs* Americans) and significantly higher *vs* at least one, including 14,347 (56%) significantly higher in QE7 compared to four continents (Web Resource S1). Of the remainder, 2 (<1%) were significantly lower than four continents (see Web Resource S1 for full list of all combinations assessed). These were two intronic SNPs in the gamma aminobutyric acid B receptor 1 (GABBR1) gene linked to nicotine dependence [15], predicted to influence alternative splicing [16]. These SNPs are common in the 1000G, with overall continental allele frequencies >0.64 for both SNPs in all continents (Web Resource S1).

#### Classification of the potentially deleterious SNPs significantly higher or lower in QE7

Out of 25,803 potentially deleterious SNPs significantly higher or lower than the overall frequency in at least one continent, 1,853 were missense nonsynonymous SNPs predicted to be potentially deleterious by SIFT or PolyPhen2 (Figure 1C). Of these potentially deleterious SNPs 1,841 (99% of 1,853) were observed in ≥1 of the 14 QE7 alleles (Table 4), and 135 were observed in ≥6 of the 14 QE7 alleles, (7% of 1,853). The total potentially deleterious missense coding SNPs identified in QE7 that are predicted significantly different in allele frequencies in Qataris compared to world populations were subclassified using databases of disease and metabolism as: (1) not previously associated with nor within a health related gene (1,336, 72% of 1,853 in ≥1 of the 14 QE7 alleles; 96, 5% of 1,853 in ≥6 of QE7); (2) in a gene previously linked to human health, but a different SNP than previously reported (442, 24% of 1,853 in ≥1/14; 30 (2% of 1,853) in ≥6/14 alleles); and (3) the gene and SNP have previously been linked to human health (63, 3% of 1,853 in ≥1/14; 9, 0.5% of 1,853 in ≥6/14 alleles). An additional 5 potentially deleterious missense SNPs were not observed to be segregating in Qatar but were observed in other continental populations a significantly higher number of times, for a total of 510 potentially deleterious missense coding SNPs in a gene previously linked to human health (Figure 1D). A further subset of these SNPs were validated by Affymetrix 5.0 genotyping in n = 149 Qatari and by TaqMan genotyping in n = 86 Qatari.

**Table 4:** Predicted Deleterious SNPs in Known Health-associated Genes Enriched in Qatari Exomes Compared to Worldwide Populations and Validated by TaqMan PCR in a Larger Qatari Population^1^.

Gene^2^		SNP^3^							Qatari allele frequency		1000 Genomes allele frequency^8^			
Symbol	Name	Amino acid substitution	dbSNP rsID	Chr	Position	Ref> alt	Risk allele	Health-associated phenotype^4,5^	QE7 exome sequencing (n = 14 alleles)^6^	QT86 TaqMan genotyping (n = 172 alleles)^7^	Europe	Asia	Africa	America
BMP4	Bone morphogenetic protein IV	Val152Ala	rs17563	14	54417522	A>G	G	Osteoporosis	0.64	0.41	0.56	0.27	0.19	0.34
ZNF229	Zinc finger protein 229	Gly662Arg	rs1434579	19	44932972	C>T	T	Resistance to tuberculosis	0.57	0.21	0.36	0.32	0.03	0.22
ULK4	UNC-51-like kinase 4	Lys569Arg	rs3774372	3	41877414	T>C	C	Diastolic blood pressure	0.57	0.34	0.20	0.15	0.23	0.15
AKAP13	A kinase anchor protein 13	Gly624Val	rs745191	15	86123170	G>T	A	Familial breast cancer	0.43	0.27	0.31	0.02	0.01	0.19
FMO2	Flavin monooxygenase II	Ser195Leu	rs2020862	1	171168584	C>T	C	Pulmonary disease	0.71	0.51	0.25	0.48	0.39	0.38
COL4A3	Collagen type IV, alpha-3	Asp326Tyr	rs55703767	2	228121101	G>T	G	Keratoconus	0.43	0.21	0.23	0.11	0.01	0.14
UTS2	Urotensin II	Thr21Met	rs228648	1	7913430	G>A	G	Type 2 diabetes	0.79	0.71	0.58	0.32	0.46	0.44
ACAT2	Acetyl-CoA acetyltransferase 2	Lys211Arg	rs25683	6	160196343	A>G	*	Plasma lipid levels	0.64	0.48	0.56	0.15	0.14	0.49
TTC37	Tetratricopeptide repeat domain 37	Arg1296Ser	rs2303650	5	94826655	C>A	*	Trichohepatoenteric syndrome	0.71	0.28	0.17	0.13	0.38	0.24
PDZRN4	PDZ domain containing ring finger 4	Gly171Ser	rs285584	12	41946539	G>A	*	Multiple sclerosis	0.57	0.22	0.16	0.11	0.24	0.14

1 Analysis of the QE7 exomes (n = 14 alleles) identified 1,841 predicted deleterious SNPs observed in at least 1 of 14 QE7 alleles and significantly different in prevalence compared to overall continental populations as represented by the 1000 Genomes. Of these, 135 SNPs were significantly different in prevalence compared to the 1000 Genomes and observed in at least 6 of 14 QE7 alleles. Of these, 39 were either in a gene previously identified within a health-linked gene (n = 9) or in a gene previously linked to human health, but a different SNP than previously reported (n = 30). Listed in this table are the 10 examples linked to diseases relevant to Qatar. SNPs from this list for which there is literature supporting a link to human health; the first 7 are from the category of “previously identified within a health-linked gene” and the last 3 are from the category of “a gene previously linked to human health, but a different SNP than previously reported.” These 10 genes were validated by TaqMan PCR in an independent group of 86 Qataris (QT86, 172 alleles, including 82 Qatari overlapping with the QA149 and 4 non-overlapping).

2 Gene symbol and name obtained from the Consensus Coding Sequence (CCDS) NCBI database [45], amino acid substitution position and residues obtained from dbSNP when available, otherwise SIFT online webserver [46].

3 SNP information includes chromosome amino acid substitution, dbSNP build 134 rsID if available, chromosome, position in GRCh37 human reference genome assembly, reference and alternate allele in QE7. Ref = references; alt = alternative; * = risk allele could not be determined.

4 Phenotype information from OMIM [12], HGMD [37], PharmGKB [38] or HUGE [39] database. Shown is the risk allele, the health-associated phenotype and the reference(s).

5 For more details and references see Details S1.

6 Shown is the alternate allele frequency determined by TaqMan in QE7 individuals; no genotypes discordant with the QE7 exome sequences were observed.

7 Shown is the alternate allele frequency in the validation set of QT86 individuals (n = 86 Qatari, 172 alleles). Failed genotypes are accounted for in the allele frequency. For statistical comparisons of the QE7 and QT86 allele frequencies, see Figure 2.

8 Shown is the alternate allele frequency in the 1000 Genomes Phase 1 population samples (n = 1,092 including n = 379 Europeans, n = 296 Asians, n = 185 Africans and n = 242 Americans; 2,184 alleles). For details of populations included in the continental allele frequency estimates, see Methods S1.

#### Variants of potential medical relevance in Qatar

The main objective of this study is to identify variants for further study in Qatar. For this purpose a set of variants were selected to be highlighted in Table 4 based on known prevalence as recorded in the medical records of Hamad Medical Corporation. Of the 30 potentially deleterious missense coding SNPs identified in ≥6 of 14 QE7 alleles as significantly higher in QE7 compared to one or more overall continental frequencies, where the gene and SNP have been previously linked to human health, we selected 7 of medical relevance in Qatar to highlight (Table 4; BMP4, ZNF229, ULK4, AKAP13, FMO2, COL4A3 and UTS2). Among these SNPs, several are relevant to disorders common in Qataris, including type 2 diabetes (UTS2), breast cancer (AKAP13), hypertension (ULK4), and nicotine dependence (FMO2) [17]. Interestingly, ZNF229 is associated with tuberculosis resistance, and the prevalence of tuberculosis is low among Qataris.

Of the 9 potentially deleterious missense coding SNPs identified in ≥6 of 14 QE7 significantly higher in QE7 compared to one or more continents where the SNP was in a gene previously linked to human health, but the potentially deleterious SNP was different than previously reported, we selected 3 of potential medical relevance in Qatar to highlight (Table 4; ACAT2, TTC37 and PDZRN4). Of these 3 genes, ACAT2 is relevant to plasma lipid levels, a finding consistent with high cholesterol levels that are common in Qataris.

## Discussion

The inhabitants of Qatar include approximately 300 thousand Qataris and 1.4 million expatriates [18], half of whom arrived in the past decade [19]. The Qataris are comprised of 3 distinct genetic subgroups, Bedouin (Q1), Persian/South Asian (Q2) and African (Q3) [9]. Like the neighboring countries in the Middle East with closely related populations, cardiovascular disease, type 2 diabetes, obesity, lung disease, breast cancer, congenital malformations, and neurologic disorders are common in the Qatari population [19]–[24]. The Qatari population is unique in that the population is small and the rate of consanguineous marriage is high [25], leading to a high probability of shared ancestry between randomly selected individuals and longer runs of homozygosity, particularly in the Q1 [9]. Using exome sequencing, this study provides the first genome-wide insight of potentially deleterious coding, nonsynonymous SNPs in the Qatari population that may be relevant for this health profile. Our analysis focused on missense SNPs likely to have a potentially deleterious effect on protein function, as these are more likely to be functional and directly involved in the disease mechanism. Of the exome SNPs identified in at least 1 of the 14 QE7 alleles compared to the GRCh37 reference allele, 2,750 (37%) were predicted to be potentially deleterious to the expressed protein. Confirmation of 131 of these SNPs by microarray in a validation group of 149 Qataris showed a remarkable correlation, i.e., potentially deleterious missense SNPs identified by exome sequencing of only 14 alleles provided a snapshot of at least some of the potentially deleterious SNPs in the Qatari population. Among these, 2 sets of missense potentially deleterious SNPs were assessed in detail; those in a gene where both the gene and SNP have been previously linked to human health and those in a gene previously linked to human health, but a different SNP than previously reported. Remarkably, despite the fact that we sequenced the exomes of only 14 Qatari alleles, among the 30 most frequent potentially deleterious SNPs observed in Qataris that had been observed in genes in other populations, 57% (17 of 30) were in genes linked to disorders common in Qatar.

Among the Qatari potentially deleterious nonsynonymous SNPs uncovered by the QE7 exome sequencing were SNPs that were novel or were significantly different (at a higher or lower frequency) than the overall frequency in at least one continent. To identify these SNPs, we used a combination of a statistical test robust to the small effects of sample size together with an Fst statistic; the latter has been successfully applied to identify missense SNPs for adaptation to altitude in Tibetans [6] and disease SNPs in individuals of Aboriginal ancestry in array genotypes [26]. This combined approach yielded a low empirical false discovery rate, as assessed for a subset of SNPs in a larger Qatari sample, indicating that the approach was successful at identifying variation that differentiates the Qatari population from worldwide populations.

Among the 392 missense potentially deleterious SNPs not in dbSNP nor observed in 1000 Genomes and hence potentially novel in the Qatari population, one was observed in all seven Qataris, 5 were observed at least once in the three population clusters (Q1, Q2, Q3), and 6 were observed at least once in two of the three population clusters. The remaining 380 novel potentially deleterious coding nonsynonymous missense SNPs were observed in only one of the QE7. Five of these novel potentially deleterious SNPs observed in two or more Qatari populations are within genes previously linked to disease, including a Phe374Leu mutation in the copper transporter protein CUTC involved in the copper metabolism [27] homozygous for the alternate allele in all seven Qataris.

Among the missense potentially deleterious SNPs with lower or higher frequency in Qataris, there were 36 SNPs lower in allele frequency than all 1000 Genomes Project overall continental frequencies (Europe, Asia, Africa, and the Americas) and significantly lower *vs* at least one, including 1 SNP significantly lower than 3 continents, 3 significantly lower than 2 continents and 32 significantly lower than 1 continent. This list includes 2 SNPs previously linked to pigmentation of skin (OCA2 His591Arg; rs1800414) [28] and hair (SLC45A2 Lys374Phe; rs1689198) [29]. An additional 8 potentially deleterious coding nonsynonymous missense SNPs were located in genes previously linked to disease, including rs1509309, a Cys206Ser mutation in serine protease 1 (PRSS1), a gene linked to hereditary pancreatitis [30]. Of the missense potentially deleterious SNPs higher in prevalence in Qataris compared to at least 1 continent and validated by TaqMan PCR in a larger Qatari population, several were relevant to diseases of high prevalence among Qataris, including genes associated with plasma lipid levels (ACAT2) and diastolic blood pressure (ULK4), as well as a specific SNP associated with type 2 diabetes (UTS2) and a specific SNP associated with nicotine dependence (FMO2). For further details and additional references regarding these SNPs, see Details S1.

By identifying potentially deleterious polymorphisms from exome sequences of 7 randomly chosen individuals of Qatari ancestry, and by validating these polymorphisms in a larger population of Qataris using genome-wide SNP array and TaqMan analysis, we have demonstrated that at least some coding sequence variations of potential medical importance within a population can be uncovered by exome sequencing of only a small number of subjects. This strategy can be used as a screening approach to identify, with reasonable confidence, common genetic variations of potential medical importance within a population. While this cannot substitute for assessing large numbers within any given population, this study demonstrates that harnessing the power of next-generation genomics can be used to identify enriched SNPs of potential medical importance that are common in populations with reasonable confidence using a small sample. In this context, a significant subset of the health-relevant variation in large segments of world populations that have yet to be analyzed could be surveyed at minimal cost. Likewise, for global-scale genomics initiatives, many small samples that cast a wide net can complement the current approach of studying large samples of fewer populations in understanding global genomic diversity.

## Methods

### Population Classification

Human subjects were recruited and written informed consent obtained at Hamad Medical Corporation (HMC), Doha, Qatar under protocols approved by the Medical Research Center & Research Committee and the Institutional Review Board of Weill Cornell Medical College in Qatar. In a previous study, we identified the three major components of ancestry in Qatar (Bedouin, Persian/South Asian, African) based on a sample of 156 unrelated Qatari genotyped using the Affymetrix Genome-Wide SNP Array 5.0 (Affymetrix Inc., Santa Clara, CA) as members of Q1 (Bedouin), Q2 (Persian/South Asian) Q3 (African) [9] based on the major ancestry group as determined by the STRUCTURE [13] algorithm with k = 3 (Figure S1). We added to this sample an additional 2 Q2 and 3 Q3 individuals who were classified into genetic subgroups using TaqMan SNP Genotyping Assay (Life Technologies Corp., Carlsbad CA) for a panel of 48 ancestry-informative SNPs (Methods S1, Table S1, and Figure S1). Thus, the complete sample of Qataris included 102 Q1, 39 Q2 and 20 Q3. This sample was used to choose the 7 Qataris for exome sequencing, where microarray and/or TaqMan genotype data collected for the remainder were used for validation purposes.

### Selection of Subjects for Exome Sequencing

From the Qataris classified as Q1, Q2 and Q3, 7 individuals with Affymetrix 5.0 genotypes available (3 Q1, 2 Q2 and 2 Q3) were randomly selected for exome sequencing. The sample included Qataris with >55% ancestry within one cluster (Q1, Q2, or Q3; Table S2 and Figure S1). Collectively, this sample is referred to as the “seven Qatari exomes” or “QE7”.

### Exome Enrichment and Sequencing

A single exome capture library was prepared for each of the QE7 individuals using standard protocols, enriched using the Agilent SureSelect Human All Exon Kit (Agilent Technologies, Inc., Santa Clara, CA) [31], and sequenced in Illumina GAIIx (Illumina Inc., San Diego, CA) using a full lane for each exome. Reads were mapped to GRCh37 using BWA v0.5.9, mean coverage depth was verified to be >30x for all samples. The breadth of coverage of the target exome ±500 bp was high, with >96% of target bases covered with at least one read.

### Identification of SNPs

SNP genotypes were called in coding exons [32] and flanking regions using the GATK [33] framework, as outlined in the GATK “Best Practices for Variant Detection V2” Wiki for all autosomal chromosomes (see Methods S1). After application of stringent quality filters, the discordance rate was calculated between the Affymetrix 5.0 genotyping array and sequencing genotypes was 0.0055, and principal component analysis of population structure was confirmed to replicate our prior analysis using array genotypes [9] (Figure 3).

### SNP Function Annotation

Available databases, including dbSNP, GATK VariantAnnotator [33], SIFT [34] and PolyPhen2 [35], were used to collect functional annotation for SNPs, including dbSNP rsIDs, genes and transcripts overlapping SNPs, coding function, amino acid substitution, and prediction of nonsynonymous missense coding SNPs. In order to maximize the number of potentially deleterious SNPs identified, SIFT and PolyPhen2 [34], [35] classifications were combined, such that a missense SNP predicted to be potentially deleterious by either algorithm was considered potentially deleterious, a more liberal classification than CONDEL [36] scores. Disease and drug metabolism annotation of known and novel SNPs was conducted using a SQL database combining public versions of OMIM [12], HGMD [37], PharmGKB [38] and HUGE [39].

SNPs were annotated in two ways: by SNP and by gene. First, SNPs involved in disease or drug metabolism were identified by querying each database with the dbSNP rsID. For OMIM SNPs, where there are nonsynonymous missense SNPs not linked to rsIDs, the database was queried using the gene name and amino acid substitution (e.g., gene “CFTR” and substitution “Arg117His”). Second, coding SNPs within genes involved in disease or drug metabolism were identified by querying each database with the gene that contained the coding SNP.

### Affymetrix and TaqMan Validation of Identified Qatari Potentially Deleterious SNPs

Affymetrix 5.0 genotypes for the 156 Qataris (102 Q1, 37 Q2, 17 Q3; Figure S1) were used to validate Qatari population allele frequency of potentially deleterious SNPs identified in the QE7 population sample (≥1 of 14 alternate alleles). Genotypes for 131 nonsynonymous missense coding SNPs identified in the QE7 were validated to be 100% consistent with the exome genotypes at the 131 potentially deleterious SNPs. Next, the QE7 individuals were excluded from the 156 Qatari and allele frequency was calculated for the 131 SNPs in the remaining 149 Qatari (QA149 = 99 Q1+35 Q2+15 Q3). In order to determine the correlation between QE7 and QA149 allele frequency, SNP allele frequency was plotted and fitted to a linear regression model, where correlations and 95% confidence interval for the allele frequency comparison were calculated. Whereas the Affymetrix 5.0 array was used to validate the 131 potentially deleterious SNPs in 149 Qataris, TaqMan PCR genotyping was used to validate 10 significantly higher SNPs predicted to be potentially deleterious by SIFT or PolyPhen2 with a known role in human health in a population sample of 86 Qataris.

### Identification of SNPs at Significantly Different Allele Frequency in Qatari Compared to Continental Populations

To identify known SNPs that have a significantly different allele frequency in the Qatari population, all known SNPs were tested for cases where the alternate allele was at a higher or lower count than expected in the 7 Qatari exomes (n = 14 alleles), when compared to the 1000 Genomes allele frequencies across populations within Europe, Asia, Africa or America. While the approach of comparing to the overall frequency in populations within a continent is not expected to identify differences between Qatari and all populations within a continent, particularly for alleles with high variance across a continent, this approach is expected to identify differences between Qatari and at least a subset of continental populations, a strategy that balances comparison to a larger sample number while flagging alleles of potential interest in the Qatari population. The allele frequency estimates used in the frequency enrichment test were calculated from the 1000 Genomes SNPs in the October 2011 Integrated Phase 1 Variant Set Release of genotypes on 1,092 individuals (ftp://ftp-trace.ncbi.nih.gov/1000genomes/ftp/release/20110521/). Individuals were placed in 4 continental groups (Table S3, Figure S2 and Figure 3; Europe, Asia, Africa or the Americas).

For each SNP discovered in the Qatari population by exome sequencing where a global allele frequency estimate in 1000 Genomes individuals (n = 1,092) could be calculated, two approaches were used to assess whether the alternative allele was at a higher or lower frequency than expected when compared to the European, Asian, African and American continental 1000 Genomes allele frequency. First, an Fst statistic was calculated [40]. Second, a binomial null sampling distribution was assumed with parameter p equal to the continental 1000 Genomes frequency and a p value was used to assess significance for a one-tailed test of the observed exome allele counts *vs* the more extreme possible frequency. Two separate one-tailed tests were conducted, the first to identify variants at significantly higher allele frequency and a second to identify variants at a significantly lower allele frequency. This testing approach was favored over Fisher’s exact test, which tends to be conservative for the comparison of proportions [41], and parametric proportion tests, since the sample size of the QE7 is small. To select SNPs, a cutoff was enforced for both statistics to harness strengths of Fst, which accounts for sampling variation in the 1000 Genomes continental allele frequency estimates [42], and the robustness of the binomial test to small counts. For the SNPs that passed both cutoffs, these were collectively referred to as “enriched” in the Qatari population, indicating that there was evidence that the alleles are at a significantly higher or lower frequency in the population of Qatar when compared to at least one continent.

This combined approach was used to assess 126,924 SNPs, where a threshold of Fst >0.25 [43] was applied to each continental comparison group and where a q-value false discovery rate (FDR) was used to assess significance for the binomial test [44], applying a threshold of FDR <0.05, again to each continental group separately. The set of significant SNPs significantly different *vs* at least one continental population (FDR <0.05 and Fst >0.25) was combined into the full list of SNPs at significantly different allele frequency.

## Supporting Information

Figure S1 Population structure analysis of 156 Qatari genotyped on the Affymetrix Genome-Wide SNP Array 5.0.
(DOC)

Figure S2 Population structure analysis of 1,092 exomes from Europe, Asia, Africa and the Americas genotyped on 48 SNPs designed to distinguish Qatari of Bedouin, Persian/South Asian or African ancestry.
(PDF)

Table S1 SNPs used to identify the 3 Qatari Genetic Subpopulations.
(PDF)

Table S2 Ancestry proportion, sequencing and coverage of seven Qatari exomes.
(PDF)

Table S3 Population samples from 1000 Genomes Project Phase 1.
(PDF)

Table S4 Allele frequency correlation between small and large samples.
(PDF)

Details S1 Supplemental Details Regarding Predicted Deleterious Nonsynonymous Coding SNPs Identified in the Qatari Population.
(PDF)

Methods S1 (PDF)

References S1 (PDF)

Web Resource S1 SNPs genotyped in seven Qatari exomes.
(PDF)
