# Supplementary material for: Exome Sequencing of Only Seven Qataris Identifies Potentially Deleterious Variants in the Qatari Population
Source: PLoS One. 2012 Nov 6;7(11):e47614. doi: 10.1371/journal.pone.0047614 (PMC3490971; doi:10.1371/journal.pone.0047614)
Supplement: Figure S1 — Population structure analysis of 156 Qatari genotyped on the Affymetrix Genome-Wide SNP Array 5.0. (DOC) [file pone.0047614.s001.doc]

**Figure S1.** Population structure analysis of 161 Qatari, including 156 genotyped on the Affymetrix Genome-Wide SNP Array 5.0 [1] and 5 genotyped on 48 SNPs able to reproduce the Q1, Q2, Q3 classification based on Affymetrix 5.0 genotypes. Shown is a plot of individual admixture component in the Bedouin, Persian, and African population clusters identified using STRUCTURE [2] with k=3. Individuals were assigned to one of three clusters (Q1 Bedouin, Q2 Persian, Q3 African) based on the cluster with the highest ancestry proportion for that individual. The origin of the three clusters (Bedouin, Persian / South Asian, or African) was determined from overlap with Human Genome Diversity Project individuals in principal components analysis and by an admixture analysis [1]. Seven individuals were selected for exome sequencing, marked with an asterisk, including individuals with >55% admixture in one cluster. Individuals are sorted from left-to-right by classified cluster, then by decreasing proportion of Bedouin admixture. Colors correspond to the three groups: Bedouin (red), Persian (green), African (blue).
