# Supplementary material for: Exome Sequencing of Only Seven Qataris Identifies Potentially Deleterious Variants in the Qatari Population
Source: PLoS One. 2012 Nov 6;7(11):e47614. doi: 10.1371/journal.pone.0047614 (PMC3490971; doi:10.1371/journal.pone.0047614)
Supplement: Figure S2 — Population structure analysis of 1,092 exomes from Europe, Asia, Africa and the Americas genotyped on 48 SNPs designed to distinguish Qatari of Bedouin, Persian/South Asian or African ancestry. (PDF) [file pone.0047614.s002.pdf]

Figure S2. Population structure analysis of 1,092 genomes from 4 continents (Table S3) using 48 SNPs designed for assigning Qatari to Q1, Q2, Q3 population clusters (Table S1). Shown is a plot of individual admixture component identified using STRUCTURE [2] with k=3. Individuals are sorted from left to right by population. Colors correspond to the major ancestry component within the three major groups: European (red), Asian (green), African (blue).

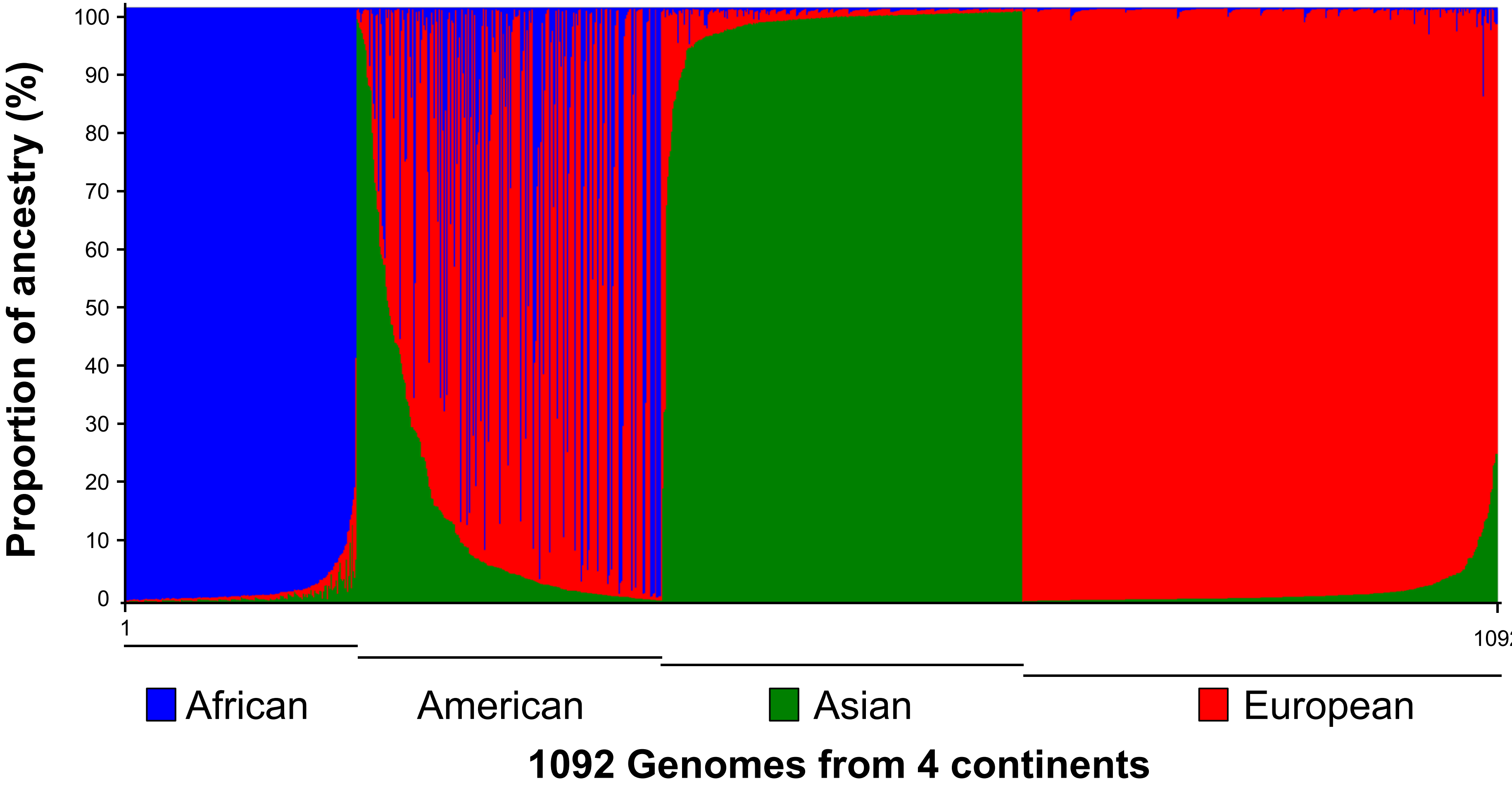
Proportion of ancestry (%)
100
90
80
70
60
50
40
30
20
10
0
1
1092
African
American
Asian
European
1092 Genomes from 4 continents

Figure S2
