# Supplementary material for: Exome Sequencing of Only Seven Qataris Identifies Potentially Deleterious Variants in the Qatari Population
Source: PLoS One. 2012 Nov 6;7(11):e47614. doi: 10.1371/journal.pone.0047614 (PMC3490971; doi:10.1371/journal.pone.0047614)
Supplement: Table S1 — SNPs used to identify the 3 Qatari Genetic Subpopulations. (PDF) [file pone.0047614.s003.pdf]

Table S1. SNPs Used to Identify the 3 Qatari Genetic Subpopulations[1]

Chromosome	Pos	dbSNP	Ref	Function	Taq Man Assay	Strand	Alleles
1	9427796	rs11121390	G	untranslated-3	C__32056208_10	+	G/T
1	13837629	rs2940305	C	intron	C___1556667_10	+	A/C
1	36107510	rs676614	C	near-gene-5	C__11182717_10	-	G/T
1	158887208	rs2106096	C	unknown	C__11334336_10	+	C/T
1	192919045	rs842806	A	unknown	C___8351682_20	-	G/T
1	192925329	rs842784	C	unknown	C___8343722_10	+	C/T
1	192927694	rs842789	T	unknown	C___8348330_10	+	C/T
1	192933397	rs1769061	T	unknown	C__26369579_20	-	G/T
1	225665694	rs6676201	T	unknown	C__11575560_10	+	A/T
1	225716241	rs7555139	G	intron	C__27148584_10	+	A/G
1	225812911	rs2639703	T	intron	C__27148635_10	+	C/T
1	226013355	rs1877724	C	intron	C__11638771_1_	+	C/T
2	6193597	rs6756093	C	unknown	C__29413282_10	+	A/C
2	55210856	rs2588515	C	intron	C___2128452_20	+	C/T
2	123327772	rs17008070	T	unknown	C__34110098_10	+	A/T
2	136910273	rs6715785	G	unknown	C__29452825_10	+	A/G
2	217920510	rs10198784	A	unknown	C___1385324_20	+	A/G
2	223843894	rs10189760	T	unknown	C___2124265_10	+	C/T
3	123404803	rs820372	T	intron	C___9532967_10	-	A/G
3	169990410	rs2140825	T	intron	C__15826452_10	-	A/G
3	179084046	rs7355960	C	intron	C__31058555_10	+	C/T
4	1334721	rs13117476	T	unknown	C__11282330_10	+	C/T
4	106145430	rs7700148	G	intron	C__33251969_10	+	A/G
4	106158216	rs3796927	G	coding-synon	C__25743542_10	-	A/G
4	156124543	rs2880416	C	unknown	C__16080280_10	-	C/G
5	36359911	rs7731311	A	unknown	C__29995760_10	+	A/C
5	122032238	rs17149518	G	unknown	C__33152018_10	+	A/G
6	3887339	rs9328202	C	unknown	C__30190362_10	+	A/C
6	22017738	rs1928168	T	unknown	C___1416263_10	-	A/G
6	102159413	rs17062374	G	intron	C__34743744_20	+	A/G

Table S1. SNPs Used to Identify the 3 Qatari Genetic Subpopulations[1] (Cont. page 2)

Chromosome	Pos	dbSNP	Ref	Function	Taq Man Assay	Strand	Alleles
6	151373655	rs9371491	A	intron	C__30326028_10	+	A/T
6	170374026	rs9460193	C	unknown	C___1466724_10	+	C/T
7	99240179	rs10242455	A	unknown	C__29897826_10	+	A/G
7	103538402	rs3905915	G	intron	C___7594821_10	+	A/G
9	19646677	rs10118808	C	intron	C__30477469_10	+	C/G
11	21025805	rs1400383	T	intron	C___8887269_10	+	A/T
11	34772501	rs7952315	T	unknown	C___1570642_10	+	A/T
11	113049287	rs7111410	C	intron	C__29205300_10	+	C/T
11	124653926	rs10466604	G	intron	C___1326067_10	+	C/G
12	20009179	rs1003189	C	unknown	C__26096839_10	+	C/T
13	22647445	rs1885895	C	unknown	C__11407211_20	+	C/T
13	72387833	rs9564847	T	intron	C__32086731_10	+	A/T
13	81562924	rs4536348	T	unknown	C_____72576_10	+	C/T
15	72552243	rs16956634	G	intron	C__32662022_10	+	C/G
15	72595571	rs12914971	A	intron	C___2900798_10	+	A/T
15	72600493	rs2959925	A	intron	C___2900791_10	-	A/C
16	6604090	rs11077049	G	intron	C___1225113_10	+	A/G
18	31632648	rs4258695	T	intron	C__26394825_20	+	G/T

[1] SNPs determined to be effective at classifying Qatari individuals as Q1, Q2 or Q3. These SNPs were selected based on maximum Q1 versus Q2 Fst, followed by maximum Fst between Q3 and the combined Q1+Q2 group for n=156 Qatari genotyped on the Affymetrix 5.0 array (see Methods for additional details on selection and validation). Shown is the chromosome, position on GRCh37 reference genome, dbSNP rsID, reference allele, function of the SNP based on location, TaqMan assay ID, strand genotyped by TaqMan assay, alleles reported by TaqMan assay.
