# Supplementary material for: Exome Sequencing of Only Seven Qataris Identifies Potentially Deleterious Variants in the Qatari Population
Source: PLoS One. 2012 Nov 6;7(11):e47614. doi: 10.1371/journal.pone.0047614 (PMC3490971; doi:10.1371/journal.pone.0047614)
Supplement: Table S2 — Ancestry proportion, sequencing and coverage of seven Qatari exomes. (PDF) [file pone.0047614.s004.pdf]

Table S2. Qatari Population Cluster Ancestry Proportions of Samples Selected for Targeted Exome Sequencing[1]

Sample information[2]			Proportion of ancestry in population cluster[3]			Mapping to target exons ± 500bp [4]			Coverage[6]	
Qatari population cluster	Sample ID (Flowcell_Lane)	Gender	Bedouin Q1	Persian/ South Asian Q2	African Q3	Read length	Reads mapped	Bases mapped	Depth	Breadth[7]
Q1	Q1_1 (101215_8)	M	0.97	0.023	0.007	124	16,221,148	2,011,422,389	64	31,428,475
Q1	Q1_2 (101215_7)	M	0.998	0.001	0.001	124	9,428,965	1,169,191,657	37	31,599,775
Q1	Q1_3 (100830_6)	F	0.668	0.319	0.013	105	13,274,630	1,393,836,173	44	31,678,095
Q2	Q2_1 (101215_6)	F	0.41	0.589	0	124	9,263,742	1,148,704,026	37	31,046,055
Q2	Q2_2 (100830_5)	F	0.41	0.584	0.006	105	13,551,738	1,422,932,508	45	31,620,722
Q3	Q3_1 (101215_5)	M	0.07	0.021	0.909	124	7,674,052	951,582,502	30	31,719,417
Q3	Q3_2 (100830_3)	F	0.175	0.271	0.555	105	14,411,946	1,513,254,319	48	31,526,132
	Mean		0.52	0.26	0.21	116	11,975,174	1,372,989,082	44	31,516,953

[1] Individuals selected for sequencing from three Qatari population clusters, including n=3 from Q1, n=2 from Q2, and n=2 from Q3; see Figure S1.

[2] Sample information includes population cluster, sample id, flowcell and lane id , gender.

[3] Proportion of Q1, Q2, and Q3 admixture determined using STRUCTURE [2]. Samples ordered from top-to-bottom by population cluster, then by decreasing proportion of Bedouin (Q1) admixture (see text re nomenclature).

[4] One paired-end Illumina library was generated for each exome enriched using the Agilent SureSelect 30MB hybrid capture [4] in a single Illumina GAIIx lane. Samples were sequenced in two separate flow cells, the first run of paired-end read length 124 bp and the second of paired-end read length 105 bp. An average of 27 million paired-end reads were sequenced per exome and an average of 28 million were mapped to the GRCh37 human reference genome assembly using BWA 0.5.9 [5] with mapping parameters "-q 15 -t 8 -o 1 -k 2 -i 15 -e -1 -l 32" and a maximum insert size of 1,000. Duplicate reads were removed using SAMtools 0.1.18 [6]. Reads mapping within 500 bp of a target exon were extracted. Shown is the read length, the number of reads mapped after filtering within 500bp +/- of a target exon, and the number of bases mapped (read length X number of reads).

[5] Coverage depth after quality filtering was compared for each sequenced exome using GATK [106]. Quality filtering included (in order) removal of reads not mapping in proper pairs, removal of duplicate reads, removal of reads mapped beyond 500 bp ± of a target exon. Additional quality filtering included identification of and realignment across indels, recalibration of base quality scores, clipping of read ends with quality below 5 and reads with mapping quality 0. Only bases with quality above 20 in reads with mapping quality above 0 were counted in the coverage analysis. Coverage depth (bases mapped / sequence length) in coding exons as defined by the Consensus Coding Project (CCDS [8]). Coverage breadth is an estimate of the sequenced exome size in bp, defined here as bases mapping to target exons ± 500 bp divided by depth.
