# Supplementary material for: Exome Sequencing of Only Seven Qataris Identifies Potentially Deleterious Variants in the Qatari Population
Source: PLoS One. 2012 Nov 6;7(11):e47614. doi: 10.1371/journal.pone.0047614 (PMC3490971; doi:10.1371/journal.pone.0047614)
Supplement: Table S3 — Population samples from 1000 Genomes Project Phase 1. (PDF) [file pone.0047614.s005.pdf]

Table S3. Population Samples from 1000 Genomes Project Phase 1[1]

Continent of origin	Population description	Code	n	2n
Europe	TSI + CEU + GBR + FIN	EUR	379	758
	Toscani in Italy	TSI	98	196
	Finnish from Finland	FIN	93	186
	British from England and Scotland	GBR	89	178
	Utah residents (CEPH) with Northern and Western European ancestry	CEU	85	170
	Iberian in Spain	IBS	14	28
Asia	CHS + CHB + JPT	ASN	286	572
	Han Chinese South	CHS	100	200
	Han Chinese in Beijing, China	CHB	97	194
	Japanese in Tokyo, Japan	JPT	89	178
Africa	YRI + LWK	AFR	185	370
	Luhya in Webuye, Kenya	LWK	97	194
	Yoruba in Ibadan, Nigeria	YRI	88	176
The Americas	ASW + MXL + PUR	AMR	242	484
	Mexican Ancestry in Los Angeles, CA	MXL	66	132
	African Ancestry in Southwest US	ASW	61	122
	Colombian in Medellin, Colombia	CLM	60	120
	Puerto Rican in Puerto Rico	PUR	55	110
Total	TSI + CEU + GBR + FIN + IBS + CHS + CHB + JPT + YRI + LWK + ASW + MXL + CLM + PUR	ALL	1,092	2,184

[1] In order to compare allele frequency between Qatari and 1000 Genomes Project populations, individuals from 12 populations sampled in four continents (Europe, Asia, Africa, America) by the 1000 Genomes Project Consortium were clustered by continent of origin. The analysis includes genotypes in exonic SNPs for (n=1,092) individuals included in the October 2011 Integrated Phase 1 Variant Set Release. Individuals were grouped by continent of origin (Europe, Asia, Africa, America). Shown is the continent, name, origin, code and size (individuals and alleles) for each population. From top-to-bottom continents and populations within each continent are ordered by decreasing sample size. Total (n=1,092) shown on bottom line.
