# Supplementary material for: Exome Sequencing of Only Seven Qataris Identifies Potentially Deleterious Variants in the Qatari Population
Source: PLoS One. 2012 Nov 6;7(11):e47614. doi: 10.1371/journal.pone.0047614 (PMC3490971; doi:10.1371/journal.pone.0047614)
Supplement: Table S4 — Allele frequency correlation between small and large samples. (PDF) [file pone.0047614.s006.pdf]

Table S4. Allele frequency correlation between small and large samples[1]

Group[2]	Population code	Small sample	Large sample	Pearson Correlation[3]
World mix 1	CEU + CHB + YRI	7	193	0.85
World mix 2	TSI + ASW + JPT	7	193	0.86
Africa	LWK	7	48	0.88
African-American	ASW	7	48	0.88
Africa	YRI	7	48	0.89
Qatar	QTR	7	149	0.89*
Central America	MXL	7	48	0.90
Africa	AFR	7	48	0.90
Central America / Caribbean	CLM + PUR + MXL	7	193	0.90
Central America, Caribbean, African-American	AMR	7	193	0.90
Caribbean	PUR	7	48	0.90
Asia	CHB	7	48	0.91
Central America / Caribbean	CLM	7	48	0.91
America	CEU	7	48	0.91
Europe	FIN	7	48	0.91
Europe	GBR	7	48	0.91
Europe	TSI	7	48	0.91
Asia	JPT	7	48	0.91
Asia	CHS	7	48	0.91
Europe	FIN + TSI + GBR	7	174	0.92
Europe	EUR	7	193	0.92
Asia	ASN	7	193	0.92

[1] In order to compare the allele frequency between small and large samples, genotypes for 20,381 deleterious missense coding SNPs were obtained for 7 Qatari and 1,092 exomes from Europe, Asia, Africa, and the Americas (Table S3). Seven random individuals were selected from each population group the allele frequency was compared to a larger independent sample of the same group. Pearson product-moment correlation was calculated for each group.

[2] Groups include each population and continent from Table S2, as well as larger groups combining populations. Combinations include two “World mix” populations combining 3 Europeans, 2 Asians and 2 Africans, a mix of populations from Central America and the Caribbean (3 Colombians, 2 Puerto Ricans, and 2 Mexicans), and a mix of European populations (3 Finnish, 2 British and 2 Tuscans).

[3] Pearson product-moment correlation was calculated using 20,381 SNPs for 1000G populations and using 131 Affymetrix 5.0 SNPs for the Qatari(*).
