# Supplementary material for: Exome Sequencing of Only Seven Qataris Identifies Potentially Deleterious Variants in the Qatari Population
Source: PLoS One. 2012 Nov 6;7(11):e47614. doi: 10.1371/journal.pone.0047614 (PMC3490971; doi:10.1371/journal.pone.0047614)
Supplement: Details S1 — Supplemental Details Regarding Predicted Deleterious Nonsynonymous Coding SNPs Identified in the Qatari Population. (PDF) [file pone.0047614.s007.pdf]

Supplemental Details Regarding Predicted Deleterious Nonsynonymous Coding SNPs Identified in the Qatari Population

SNPs Where both the SNP and the Gene Have been Previously Demonstrated to be of Potential Medical Importance (Table II). Among the SNPs identified in the QE7 exome analysis where both the SNP and gene have been previously reported, several were relevant to diseases of high prevalence among the Qatari population, including type 2 diabetes, coronary artery disease and hypertension.

PPARG. The peroxisome proliferator-activated receptor gamma (PPARG) gene is a nuclear receptor that regulates fatty acid storage and glucose metabolism. PPARG associated variations have been linked to type 2 diabetes in a large number of studies, as well as to obesity, atherosclerosis and cancer [26]. The Pro12Ala variation observed in the QE7 population has been linked to an increased insulin sensitivity / and decreased risk of developing hyperglycemia [27–31], preterm birth [32], and colorectal carcinoma [33].

PON2. The paraoxonase gene (PON2) codes for a ubiquitously expressed membrane based protein thought to function as an antioxidant [34]. Mutations in PON2 have been associated with cardiovascular disease and diabetes [35,36]. In association with smoking, the Ser311Cys variation has been linked to an increased risk for myocardial infarction [37], and for type 2 diabetes in Northern Chinese [38].

NAT2. The N-acetyltransferase (NAT2) gene codes for N-acetyltransferase, an enzyme that metabolizes arylamine, hydrazine and benzodiazepine drugs [39]; the Arg197Gln variant has been linked to slow metabolism of clonazepam [40].

MTR. The 5-methyltetrahydrofolate-homocyteine methyltransferase (MTR) gene codes for methionine synthase, which converts homocysteine to methionine [41]. MTR mutations are linked

to homocystinuria, neural tube defects, pseudoachondroplasia and Down syndrome [42], with the Asp473Gly variant associated with cardiovascular disease [43].

NQO1. The NAD(P)H: quinone oxidoreductase 1 (NQO1) codes for cytosolic enzyme that catalyzes quinoid compounds into the less toxic hydroquinones [44]. Variations in NQO1 have been associated with leukemia, lung and bladder cancer [45–47], and the Pro187Ser variation with colorectal cancer [48], increased risk of benzene poisoning [49] and poor survival in breast cancer treated with chemotherapy [50].

ULK4. Variations in the UNC-51-like kinase 4 (ULK4) gene has been linked to blood pressure control in large studies, and the Lys569Arg variation is associated with an increase in diastolic blood pressure [51].

CDC6. The cell division control protein homolog (CDC6) is essential for the initiation of DNA replication; the Val441Ile has been linked to increased rate of FEV1 decline in ex-smokers with COPD [52].

PARP1. Poly[ADP-ribose] polymerase (PARP1) modifies nuclear proteins by poly ADP-ribosylation, and functions in DNA damage repair [53]. PARP1 variations have been associated with type 1 diabetes, Fanconi anemia and gastric cancer; the Val762Ala variation has been associated with increased risk of cancer in Asians and decreased risk of cancer in Europeans [54].

BDNF. Brain-derived neurotrophic factor (BDNF) is a member of the neurotrophin family of growth factors, a groups of genes with widespread function in the CNS and in other organs [55]. The Val66Met variation is the best known BDNF variant, associated with alterations in brain anatomy and memory [56,57]. When mice with the homozygous Val66Met variant are placed in stressful settings, they exhibit increased anxiety-related behaviors. In humans, this variation is thought to mediate a predisposition to anxiety and depressive disorders and influences motor system function [56,57].

PPP1R3A. Protein phosphatase 1 regulatory subunit 3A (PPP1R3A) codes for a form of protein phosphase 1 that binds to muscle glycogen with high affinity. Mutations in PPP1R3A have been linked to vascular disease and diabetes phenotypes [58]. The Asp905Tyr variation has been associated with type 2 diabetes and insulin sensitivity [59].

SNPs Where the Gene, but not the SNP, Have Been Previously Demonstrated to be of Potential Medical Importance (Table III). This group of SNPs discovered by exome sequencing of QE7 represented SNPs where the gene has been previously linked to human health, but a different deleterious SNP than previously reported. Several are relevant to diseases of high prevalence among the Qatari population, including neurologic disorders.

HMCN1. Hemicentin (HMCN1) is an extracellular member of the immunoglobin superfamily, thought to function in the organization of hemidesmosomes [60]. Although controversial, mutations in HMC1 have been linked to age-related macular degeneration [61–63].

IκB-KAP. Mutations in inhibitor kappa light polypeptide gene enhancer in B-cells, kinase associated protein (IκB-KAP) have been linked to familial dysautonomia [64,65].

VSX1. Visual system homeobox gene 1 (VSX1) functions as a transcription regulator, binding to the locus control region of the red/green visual pigment cluster. The VSX1 protein may regulate off-center cone bipolar cell development [66]. Variations in the VSX1 gene are linked to keratoconus and polymorphous corneal dystrophy [67].

EVC. Mutations in the Ellis-van Creveld (EVC) gene are linked to the dwarfism and heart defects that characterize the Ellis-van Creveld syndrome and one mutation in the EVC gene has been associated with Weyers acrodental dysostosis, a recessive disorder characterized by chondrodysplasia, polydactyly, nail dysplasia, orofacial abnormalities and sometimes cardiac defects [68].

SGCG. Variations in the gamma-sarcoglycan (SGCG) gene, a sarcolemmal transmembrane glycoprotein that interacts with dystrophin, are linked to limb girdle muscular dystrophy [69].

SACS. The sacsin (SACS) gene is expressed in the central nervous system, skin and skeletal muscles; mutations in SACS can cause autosomal recessive Charlevoix-Saguenay spastic ataxia, a neurodegenerative disorder characterized by cerebellar ataxia, spasticity and peripheral neuropathy [70].

OSMR. The oncostatin-M specific receptor subunit beta (OSMR) gene functions as a component of for interleukin 31 and mutations of OSMR are linked to primary cutaneous amyloidosis [71].

ARHGEF10. The rho guanine nucleotide exchange factor 10 (ARHGEF10) gene is a rho GTPase; variations in ARHGE10 are associated with slow conduction and thin myelination of peripheral nerves [72].

CACNA1S. The calcium channel, voltage-dependent, L-type, alpha 1S subunit (CACNA1S) gene encodes 1 of the 5 subunits of a calcium channel in skeletal muscle. Mutations in CACNA1S have been associated with hypokalemic periodic paralysis, thyrotoxic periodic paralysis and malignant hyperthermia [73,74].

RSPH4A. Radial spoke head protein 4 homolog A (RSPH4A) codes for a homolog of the radial spokes of cilia in *Chlamydomonas reinhardtii* [75]. Mutations in RSPH4A are linked to primary ciliary dyskinesia [76].

Predicted Deleterious SNPs in Known Health-associated Genes Enriched in Qatari Exomes Compared to the Worldwide Populations (Table IV). Among the SNPs enriched in the Qatari population include disorders relevant to diseases of high prevalence among Qataris, including resistance to tuberculosis, breast cancer, hypertension, diabetes and dyslipidemia.

BMP4. The Val152Ala variant of bone morphogenic protein 4 (BMP4), a member of a family of growth factors critical for morphogenesis [77], has been linked to osteoporosis. Postmenopausal women homozygous for the C (alanine) allele have lower total and intertrochanteric hip bone mineral density [78].

ZNF229. The zinc finger protein 229 (ZNF229) is a transcription factor; individuals with the Gly662Arg variant have a higher resistance to tuberculosis [79], and is predicted by the SMART algorithm [13] to be located within the predicted zinc finger binding domain. Consistent with this observation, while tuberculosis is common in non-Qataris living in Qatar, it is infrequent in Qataris [80–82]. Only 3% of registered tuberculosis cases in the 2003-2010 period were Qatari, *vs* 40% Nepali and 20% Indian [83].

ULK4. Three nonsynonymous SNPs in ULK4 (unc-51-like kinase 4) have been associated with diastolic blood pressure in a genome-wide association study (GWAS) of 29K individuals of European ancestry [51]. The association was validated in a larger study of 200K individuals of European ancestry [51] for the rs3774372 SNP. The allele frequency in Q1 Qatari of Arabian ancestry is highest in the world for the risk allele (C) at 0.24 allele frequency, shown in the ICBP-GWAS study to increase diastolic blood pressure by 0.367 mmHg per copy ($p=9x10^{-14}$). The Lys569Arg ULK4 variation observed in Qataris was validated by Affymetrix 5.0 microarray (Table II). The allele frequency in Qatari populations was also validated by TaqMan genotyping and was 0.24 in Q1, 0.18 in Q2, 0.18 in Q3, *vs* 0.19 in Europeans from 1000G, 0.14 in Asians from 1000G and 0.22 in Africans from 1000G. In the ICBP-GWAS study, the reported allele frequencies were also lower than Q1 for HapMap Central European (0.24), HapMap Chinese/Japanese (0.15), HapMap Yoruban (0.22), East Asian (0.15), South Asian (0.17) and African American (0.22) populations. These SNPs are good candidates for contributing to the inci-

dence of high diastolic blood pressure in Qatar, based on high linkage disequilibrium between the 2 enriched SNPs and the 4 associated SNPs in ULK4 [http://hapmap.ncbi.nlm.nih.gov/].

AKAP13. A-kinase anchor protein 13 (AKAP13) binds to the regulatory subunit of protein kinase A, and functions in GTPase activity [84]. The Gly624Val variant that is highly prevalent in Qataris has been linked to familial breast cancer, of interest as breast cancer is the most common cancer among Qatari women [85].

FMO2. Flavin containing monooxygenase II (FMO2) is a lung-specific xenobiotic metabolizing enzyme that is non-functional in Europeans [86] but is functional in African Americans and Hispanics[87,88]. The prevalence of the functional copy in African Americans is 26%, while the frequency in Puerto Ricans (7%) is higher than in Mexican Americans (2%) [88]. FMO2 metabolizes phenothiazines bearing C6 and C7 alkyl side chains in humans [88]. The SNP was observed in all three Qatari sub-populations, with highest allele frequency in the Q3 of African ancestry, where the 1000G population with the highest allele frequency is also the African population. The Middle East is a region with high prevalence of pulmonary disease [89,90]. Assuming the predicted deleterious SNP Ser192Leu has a role in the function of FMO2, it seems reasonable to hypothesize that the FMO2 SNP contributes to the genetic susceptibility of pulmonary disease in Qataris. In a bioinformatic prioritization meta-analysis of nicotine dependence GWAS data, FMO2 Ser192Leu was among the top 10 nicotine dependence SNPs in Europeans [91].

COL4A3. The collagen type IV, alpha 3 chain (COL4A3) is a major component of basement membranes and is an antigen target in Goodpasture syndrome [92]. Mutations in COL4A3 are associated with Alport syndrome, with kidney failure and hearing loss [93]. The Asp326Tyr variant observed in high prevalence in Qataris compared to Asians and Africans is associated with keratoconus [94].

UTS2. SNPs in UTS2 (urotensin-2) and its overlapping adjacent gene PER3 (period homolog 3)

have been associated with type 2 diabetes in multiple Asian populations including Chinese [95–98], and Iberian [99] but not Japanese [100]. The SNP is not enriched *vs* Europeans nor *vs* Africans, hence it is unlikely to explain the difference in diabetes prevalence between populations. The 1000G population with the highest allele frequency is Europeans at 0.6, lower than in Q1 and Q2.

ACAT2. Acyl-CoA cholesterol acyltransferase 2 (ACAT2) is an intracellular cholesterol esterification enzyme found in the intestine and liver [101]. Three polymorphisms, including two SNPs and one indel, have been associated with lipid levels in Singaporean ethnic groups (Chinese, Malys and Indians), although the associations were not consistent across gender nor ethnicity[102]. The potentially deleterious SNP (Lys211Arg) was identified in Qataris of Arabian (Q1) and African (Q3) but not Persian (Q2) ancestry. The Qatari major allele is the minor allele in Asians and Africans from the 1000G, although the major allele in Europeans. While associations between polymorphisms in ACAT2 and lipid levels European populations have not been established, it is reasonable to hypothesize that this gene contributes to genetic variation in lipid levels in Europe and that an influx of European haplotypes into Qatar may be contributing to the incidence of high lipid levels in the Qatari people [103].

TTC37. The SNP identified in the Qataris, while predicted to be deleterious, is different from that known to be associated with disease. Variants in the tetratricopeptide repeat domain 37 (TTC37) gene has been linked to trichohepatoenteric syndrome [104].

PDZRN4. The PDZ domain containing ring finger 4 (PDZRN4) gene has been associated with multiple sclerosis [105]. While the SNPs observed in this gene are of high prevalence in Qataris and are predicted to be deleterious, the SNPs are different from those known to be linked to disease.
