# Supplementary material for: Exome Sequencing of Only Seven Qataris Identifies Potentially Deleterious Variants in the Qatari Population
Source: PLoS One. 2012 Nov 6;7(11):e47614. doi: 10.1371/journal.pone.0047614 (PMC3490971; doi:10.1371/journal.pone.0047614)
Supplement: Methods S1 — (PDF) [file pone.0047614.s008.pdf]

# Supplemental Methods

## Population Classification

Human subjects were recruited at Hamad Medical Corporation (HMC), Doha, Qatar under HMC and Weill Cornell Medical College-Qatar Institutional Review Board approved protocols. To classify the population into the Qatari Q1, Q2 and Q3 genetic subgroups, Affymetrix Genome-Wide SNP Array 5.0 (Affymetrix Inc., Santa Clara, CA) genotypes were generated for 156 unrelated Qataris [1]. In our previous study, we identified the three major components of ancestry in Qatar (Bedouin, Persian/South Asian, African) and classified a population sample of 156 Qatari as members of Q1 (Bedouin), Q2 (Persian/South Asian) Q3 (African) [1] based on the major ancestry group as determined by the STRUCTURE [2] algorithm with k=3 (Figure S1).

To add to these 156 subclassified Qataris, we constructed a panel of 48 SNPs able to reproduce the Q1, Q2, Q3 classification based on Affymetrix 5.0 genotypes, and recruited additional individuals not genotyped by Affymetrix 5.0 microarray. A panel of 48 SNPs was selected based on maximum Fst [3] between Q1 and Q2 in the 156 Qatari, followed by maximum Fst between the combined Q1 + Q2 group and Q3 and by their ability to correctly classify random selections from the 156 Qatari with the Q1 Q2 Q3 classification masked (Table S1). Using TaqMan SNP Genotyping Assay (Life Technologies Corp., Carlsbad CA) for this panel of 48 SNPs, an additional 2 Q2 and 3 Q3 individuals not included in the sample genotyped by Affymetrix 5.0 [1] were identified by TaqMan genotyping (Figure S1). Thus, the complete 161 Qatari sample included 102 Q1, 39 Q2 and 20 Q3.

## Selection of Subjects for Exome Sequencing

From the Qataris classified as Q1, Q2 and Q3, 7 individuals with Affymetrix 5.0 genotypes available (3 Q1, 2 Q2 and 2 Q3) were selected for exome sequencing based on central loca-

tion within the principal components analysis population clusters. The sample included Qataris with >55% ancestry within one cluster (Q1, Q2, or Q3) (Figure S1, S2). Collectively, this sample is referred to as the "seven Qatari exomes" or "QE7".

**Exome Enrichment and Sequencing**

A single exome capture library was prepared for each of the QE7 individuals using standard protocols, with 38 Mb of exome DNA enriched using the Agilent SureSelect Human All Exon Kit solution hybrid selection platform (Agilent Technologies, Inc., Santa Clara, CA) [4]. Paired-end 105 or 124 bp reads were sequenced in Illumina GAIIx (Illumina Inc., San Diego, CA) using a full lane for each exome. Reads were mapped to 1000 Genomes version of GRCh37 human reference genome using BWA v0.5.9 with default parameters [5]. Unmapped reads, reads not mapping in a proper pair with insert size under 1,000 bp, reads not mapping to coding exons, and duplicate reads were removed using SAMtools [6]. Mean coverage depth was verified to be >30x for all samples, including target exome and bases within 500 bp of a target exon. The breadth of coverage of the target exome ±500 bp was high, with >96% of target bases covered with at least one read. Depth calculations were conducted using the Genome Analysis Tool Kit (GATK) DepthOfCoverage program [7].

**Identification of SNPs**

SNP genotypes were called in coding exons [8] and flanking regions using the GATK[7] framework, as outlined in the GATK "Best Practices for Variant Detection V2" Wiki for all autosomal chromosomes. The Y and mitochondrial chromosomes were not included in the analysis because no allele frequency information in 1000 Genomes populations is currently available for these chromosomes. The X chromosome was also excluded in order to compare allele frequency in mixed gender population samples, given the small sample size of n=4 males and n=3 females.

In order to maximize the quality of genotypes, a strict quality filtering protocol was applied based on recommendations in the GATK Wiki. Mapped reads for all samples were merged into a single BAM file, with each sample identified by a read group tag [6]. SNP clusters attributable to misalignment at known indels were flagged and realigned using three criteria: (1) proximity (10 bp) with a known indel in dbSNP build 134 (abbreviated elsewhere in this text as dbSNP); (2) 3 or more out of 10 bases in a window were variants, with coverage depth of at least 4; and (3) >15% of base quality sum for mismatch position. A LOD score was calculated and flagged clusters with LOD >5.0 were realigned using all reads mapped to the indel site. Read pair-mapping information was updated to reflect the indel position using the Picard 1.33 FixMateInformation function (http://picard.sourceforge.net). Base quality scores were recalibrated to minimize spurious variants due to bias attributed to run, lane, cycle number, dinucleotide, mapping quality and sequencer base quality. The recalibration procedure took the listed features as a list of input covariates, calculated the observed mismatch rate for each combination of values, compared the expected *vs* observed quality score at non-dbSNP sites, and then adjusted the quality scores for combinations with quality values that deviate from expectation [http://www.broadinstitute.org/gsa/wiki/index.php/Base_quality_score_recalibration]. Bases with a quality score below 5 before and after recalibration were clipped (assigned a CIGAR value of skip and base quality zero) [6].

Genotypes for SNPs within ± 500 bp of a coding exon were simultaneously generated for all 7 exomes using the GATK Unified Genotyper algorithm [7]. Low-quality SNPs were filtered out using the following criteria: (1) missing genotype for one or more of the 7 Qatari subjects; (2) genotype quality score <20 for each individual genotype; (3) quality <50 for the multi-sample genotype likelihood; (4) SNP clusters (3 in 10 bp); (5) overlap with a known insertion or deletion; (6) >10% of reads with mapping quality 0; (7) quality-by-depth score <5; (8) homopolymer

runs >5 bp; and (9) strand bias >-0.1. A hard minimum read depth threshold was not set, as it is not recommended for exome sequencing and the GATK quality score incorporates depth into its quality score calculation [7].

In order to obtain a high-quality call set of QE7 SNPs for comparison of allele frequency versus 1000 Genomes (1,092 individuals included in the October 2011 Integrated Phase 1 Variant Set Release of 1000 Genomes genotypes, see Table S3 for population information for the 1000 Genomes), the SNPs that remained after application of the genotyping filters described above were classified as “excluded”, “polymorphic in QE7 or 1000G”, and “polymorphic in QE7”. “Excluded SNPs” were defined as SNPs where the 1000 Genomes and QE7 alternate allele did not match (potential trialleleic SNPs), in highly polymorphic HLA genes [9] and in predominantly pseudogene olfactory receptors [10]. “Polymorphic in QE7 or 1000G” were defined as the set of SNPs that were observed at least once in QE7 or 1000G (≥1 of 2,198 alleles) that remained after filtering steps. “Polymorphic in QE7” were the subset of “Polymorphic in QE7 or 1000G” observed at least once (≥1 of 14 alleles). Alternate allele frequency and allele count was calculated for QE7 and each of the 1000G continents (for populations in European, Asian, African, or American) at sites polymorphic in QE7 or 1000G.

In order to assist with allele frequency validation and literature searches, the dbSNP rsID and Affymetrix Genome-Wide SNP Array 5.0 Probeset ID was assigned to SNPs in the call set of SNPs polymorphic in QE7 or 1000G. First, dbSNP build 134 in VCF format was queried using a Perl script to assign rsIDs for SNPs in the QE7 call set with matching chromosome, position on GRCh37 genome build, reference allele and alternate allele. Next, the set of rsIDs was used to query the Affymetrix 5.0 probeset annotation file in order to obtain the probeset id.

**SNP Function Annotation**

Available databases were used to collect functional annotation of SNPs, including dbSNP rsIDs, genes and transcripts overlapping the SNP, coding function, amino acid substitution, and prediction of nonsynonymous missense coding SNP effect using dbSNP, GATK VariantAnnotator [7], SIFT [11] and PolyPhen2 [12], and Perl scripts. Coding SNPs were identified and assigned to genes and exons using a Perl script that assigns to a gene all SNPs between the start and end positions of a coding exon with boundaries defined by the CCDS database [8]. Coding SNP type (silent, missense, 3' untranslated, 5' untranslated, nonsense, frameshift, splice donor or splice acceptor), polypeptide position and amino acid substitution was obtained from dbSNP. For SNPs not present in dbSNP, coding function was obtained from SIFT online webserver [11]. For SNPs where multiple isoforms exist and the SNP could be annotated as silent *vs* missense or non-coding *vs* missense, the missense SNP was presented. For SNPs where SIFT and dbSNP disagreed on the transcript position or amino acids, the dbSNP annotation was kept. Bioinformatics prediction of SNP function included missense deleterious effect using SIFT [11] or PolyPhen2 [12]. Protein domain presence was also determined for deleterious SNPs highlighted in tables II, III, and IV using SMART7 [13]. In order to maximize the number of deleterious SNPs identified, SIFT and PolyPhen2 [12] classifications were combined, such that a missense SNP predicted deleterious by either algorithm was considered deleterious, a more liberal classification than CONDEL [14] scores. Disease and drug metabolism annotation of known and novel SNPs was conducted using a SQL database combining public versions of OMIM [15] (18,573 records), HGMD [16] (60,489 records), PharmGKB [17] (3,447 records) and HUGE [18] (4,421 records). Databases were converted to SQL format using a Perl script, and SNPs were annotated by querying the database using SQL queries joined on SNP rsID, gene name, or gene name and amino acid substitution.

SNPs were annotated in a two ways: by SNP and by gene. First, SNPs involved in disease or drug metabolism were identified by querying each database with the dbSNP rsID. For OMIM SNPs, where there are nonsynonymous missense SNPs not linked to rsIDs, the database was queried using the gene name and amino acid substitution (e.g., gene "CFTR" and substitution "Arg117His"). Second, SNPs within genes involved in disease or drug metabolism were identified by querying each database with the gene that contained the coding SNP.

**Affymetrix Validation of Identified Qatari Deleterious SNPs**

Affymetrix Genome-Wide SNP Array 5.0 (Affymetrix Inc., Santa Clara, CA) genotypes for 156 Qatari individuals with >55% proportion of ancestry in one of the 3 Qatari population clusters (102 Q1, 37 Q2, 17 Q3; Figure S1) were used to validate Qatari population allele frequency of deleterious SNPs identified in the QE7 population sample (≥1 of 14 alternate alleles). Genotypes for 131 nonsynonymous missense coding SNPs identified in the QE7 were extracted in BRLMM format (0 = AA, 1 = AB, 2 = BB) and converted to a consistent strand format (0, 1, 2 alternate alleles) using a Perl script. Strand assignment for Affymetrix 5.0 genotypes was based on annotation file from the manufacturer specifying A and B allele. The genotypes for the QE7 was validated to be 100% consistent with the exome genotypes at the 131 deleterious SNPs. Next the QE7 individuals were excluded from the 156 Qatari and allele frequency was calculated for the 131 SNPs in remaining 149 Qatari (QA149 = 99 Q1 + 35 Q2 + 15 Q3) using a Perl script.

In order to determine the correlation between QE7 and QA149 allele frequency, SNP allele frequency was plotted and fitted to a linear regression model using R. The Pearson product-moment correlation, probability and 95% confidence interval for the allele frequency comparison was calculated using R.

**Identification of SNPs at Enriched Allele Frequency in Qatari Compared to Continents**

To identify known SNPs that have a enriched allele frequency in the Qatari population, all known SNPs were tested for cases where the alternate allele was at a higher or lower count than expected in the 7 Qatari exomes (n=14 alleles), when compared to the 1000 Genomes allele frequency in European, Asian, African or American individuals. The allele frequency estimates used in the frequency enrichment test were calculated from the 1000 Genomes SNPs in the October 2011 Integrated Phase 1 Variant Set Release of genotypes on 1,092 individuals (ftp://ftp-trace.ncbi.nih.gov/1000genomes/ftp/release/20110521/). Individuals were placed in 4 continental groups (Table S3, Figures S2, S3); Europe, Asia, Africa or the Americas). The European populations included (in order of decreasing sample size): Toscani in Italy (TSI), Finnish from Finland (FIN), British from England and Scotland (GBR), Utah residents with Northern and Western European ancestry (CEU), and Iberians in Spain (IBS), (n =379; 98 TSI + 98 FIN + 89 GBR + 85 CEU + 14 IBS). The Asian populations included: Han Chinese South (CHS), Han Chinese in Beijing (CHB) and Japanese in Tokyo, Japan (JPT); and (n=286; 100 CHS + 97 CHB + 89 JPT). The African populations included: Yoruba in Ibadan, Nigeria (YRI); and Luhya in Webuye, Kenya (LWK); (n =185; 97 YRI + 88 LWK). The fourth continental group was comprised of admixed populations originating in the Americas [19,20], including individuals of Mexicans ancestry from Los Angeles CA (MXL), individuals of African Ancestry in Southwest US (ASW), Colombians in Medellin, Colombia, and Puerto Ricans in Puerto Rico (PUR) (n=242; 66 MXL + 61 ASW + 60 CM + 55 PUR).

For each SNP discovered in the Qatari population by exome sequencing where a global allele frequency estimate in 1000 Genomes individuals (n=1,092) could be calculated, two approaches were used to assess whether the alternative allele was at a higher or lower count than expected when compared to the European, Asian, African and American continental 1000 Ge-

nomes allele frequency. First, an Fst statistic was calculated [21]. Second, a binomial null sampling distribution was assumed with parameter p equal to the continental 1000 Genomes frequency and a p value was used to assess significance for a one-tailed test of the observed exome allele counts versus the more extreme possible frequency. This testing approach was favored over Fisher's exact test, which tends to be conservative for the comparison of proportions [22], and parametric proportion tests, since the small sample size of the QE7 was small. To select SNPs, a cutoff was enforced for both statistics to harness strengths of Fst, which accounts for sampling variation in the 1000 Genomes continental allele frequency estimates [23], and the robustness of the binomial test to small counts. For the SNPs that passed both cutoffs, these were collectively referred to as "enriched" in the Qatari population, indicating that there is evidence that the alleles are at a significantly higher or lower frequency in the population of Qatar when compared to at least one continent.

This combined approach was used to assess 126,924 SNPs, where a threshold of Fst >0.25 [24] was applied to each continental comparison group and where a q-value false discovery rate (FDR) was used to assess significance for the binomial test [25], applying a threshold of FDR <0.05, again to each continental group separately. The set of significant SNPs significantly different *vs* at least one continental group (FDR <0.05 and Fst >0.25) was combined into the full list of SNPs at significantly different allele frequency.

In the data analysis, all SNPs were represented in terms of the number of alternate alleles observed and in terms of the enrichment results. The SNPs were separated into two groups based on the QE7 allele frequency threshold of >7% (≥1 in 14 alleles) and >40% (≥6 in 15 alleles). Enrichment results were further divided into 25 subcategories on the basis of the number of continents higher or lower and significantly higher or lower, where "higher" and "lower" is defined by the difference in allele frequency (QE7 minus 1000G continent), such that a negative differ-

ence indicates lower in Qatar and a positive difference indicates higher in QE7. Significantly higher or lower adds the criteria of Fst >0.25 and FDR <0.05 when comparing the observed and expected QE7 allele count. In order to quantify the number of SNPs where the Qatari allele frequency is higher or lower than 1, 2, 3 or 4 continents, a 16-category binary classification was conducted for each SNP (QE7>EUR, QE7>ASN, QE7>AFR, QE7>AMR, QE7<EUR, QE7<ASN, QE7<AFR, QE7<ASN; repeated for higher or lower and for significantly higher or lower), and the frequency of each combination observed at least once was counted and summarized using a Perl script.

**TaqMan Validation of Identified Qatari Deleterious SNPs**

Whereas the Affymetrix 5.0 array was used to validate the 131 deleterious SNPs in 149 Qataris, TaqMan PCR genotyping was used to validate 10 significantly higher SNPs (rs17563, rs1434579, rs3774372, rs745191, rs2020862, rs55703767, rs228648, rs25683, rs2303650, and rs285584) predicted deleterious by SIFT [11] or PolyPhen2 [12] with a known role in human health in a population sample of 86 Qataris (33 Q1, 35 Q2, 18 Q3) including 82 from the 149 genotyped on Affymetrix 5.0 and five Qatari (2 Q2, 3 Q3) classified using the panel of 48 ancestry-informative SNPs. Genotypes and allele frequency were also validated in the 7 Qatari genomes used for the exome sequencing. TaqMan genotyping was carried out using standard protocols. Briefly, 1 µl of diluted DNA was combined with 12.5 µl of TaqMan Genotyping Master mix, 10.25 ul DEPC-treated water and 0.625 µl 40x Genotyping Assay Mix in a 96-well plate and RT-PCR allelic discrimination was conducted using an ABI TaqMan 7500 (Life Technologies Inc, Carlsbad CA). Genotypes were assigned manually from allelic discrimination plots and reference/alternate alleles were counted.
