# Supplementary material for: Exome Sequencing of Only Seven Qataris Identifies Potentially Deleterious Variants in the Qatari Population
Source: PLoS One. 2012 Nov 6;7(11):e47614. doi: 10.1371/journal.pone.0047614 (PMC3490971; doi:10.1371/journal.pone.0047614)
Supplement: References S1 — (PDF) [file pone.0047614.s009.pdf]

Supplemental References

1. Hunter-Zinck H, Musharoff S, Salit J, Al-Ali KA, Chouchane L, et al. (2010) Population genetic structure of the people of Qatar. Am J Hum Genet 87: 17-25.

2. Pritchard JK, Stephens M, Donnelly P (2000) Inference of population structure using multilocus genotype data. Genetics 155: 945-959.

3. Weir BS, Cockerham CC (1984) Estimating *F*-statistics for the Analysis of Population Structure. Evolution 38: 1358-1370.

4. Gnirke A, Melnikov A, Maguire J, Rogov P, LeProust EM, et al. (2009) Solution hybrid selection with ultra-long oligonucleotides for massively parallel targeted sequencing. Nat Biotechnol 27: 182-189.

5. Li H, Durbin R (2010) Fast and accurate long-read alignment with Burrows-Wheeler transform. Bioinformatics 26: 589-595.

6. Li H, Handsaker B, Wysoker A, Fennell T, Ruan J, et al. (2009) The Sequence Alignment/Map format and SAMtools. Bioinformatics 25: 2078-2079.

7. DePristo MA, Banks E, Poplin R, Garimella KV, Maguire JR, et al. (2011) A framework for variation discovery and genotyping using next-generation DNA sequencing data. Nat Genet 43: 491-498.

8. Pruitt KD, Harrow J, Harte RA, Wallin C, Diekhans M, et al. (2009) The consensus coding sequence (CCDS) project: Identifying a common protein-coding gene set for the human and mouse genomes. Genome Res 19: 1316-1323.

9. de Bakker PI, McVean G, Sabeti PC, Miretti MM, Green T, et al. (2006) A high-resolution HLA and SNP haplotype map for disease association studies in the extended human MHC. Nat Genet 38: 1166-1172.

10. Menashe I, Man O, Lancet D, Gilad Y (2003) Different noses for different people. Nat Genet 34: 143-144.

11. Kumar P, Henikoff S, Ng PC (2009) Predicting the effects of coding non-synonymous variants on protein function using the SIFT algorithm. Nat Protoc 4: 1073-1082.

12. Adzhubei IA, Schmidt S, Peshkin L, Ramensky VE, Gerasimova A, et al. (2010) A method and server for predicting damaging missense mutations. Nat Methods 7: 248-249.

13. Letunic I, Doerks T, Bork P (2012) SMART 7: recent updates to the protein domain annotation resource. Nucleic Acids Res 40: D302-D305.

14. Gonzalez-Perez A, Lopez-Bigas N (2011) Improving the assessment of the outcome of nonsynonymous SNVs with a consensus deleteriousness score, Condel. Am J Hum Genet 88: 440-449.

15. Online Mendelian Inheritance in Man, OMIM. http://www.omim.org. [last accessed July 13, 2012]

16. Stenson PD, Mort M, Ball EV, Howells K, Phillips AD, et al.(2009) The Human Gene Mutation Database: 2008 update. Genome Med 1: 13.

17. Klein TE, Chang JT, Cho MK, Easton KL, Fergerson R, et al. (2001) Integrating genotype and phenotype information: an overview of the PharmGKB project. Pharmacogenetics Research Network and Knowledge Base. Pharmacogenomics J 1: 167-170.

18. Yu W, Yesupriya A, Wulf A, Hindorff LA, Dowling N, et al. (2011) GWAS Integrator: a bioinformatics tool to explore human genetic associations reported in published genome-wide association studies. Eur J Hum Genet 19: 1095-1099.

19. Choudhry S, Coyle NE, Tang H, Salari K, Lind D, et al. (2006) Population stratification confounds genetic association studies among Latinos. Hum Genet 118: 652-664.

20. Bryc K, Velez C, Karafet T, Moreno-Estrada A, Reynolds A, et al. (2010) Colloquium paper: genome-wide patterns of population structure and admixture among Hispanic/Latino populations. Proc Natl Acad Sci U S A 107 Suppl 2: 8954-8961.

21. Akey JM, Zhang G, Zhang K, Jin L, Shriver MD (2002) Interrogating a high-density SNP map for signatures of natural selection. Genome Res 12: 1805-1814.

22. Berkson J (1978) In Dispraise of the Exact Test. Do the Marginal Totals of the 2x2 Table Contain Relevant Information Respecting the Table Proportions? Journal of Statistical Planning and Inference 2: 27-42.

23. Holsinger KE, Weir BS (2009) Genetics in geographically structured populations: defining, estimating and interpreting F(ST). Nat Rev Genet 10: 639-650.

24. Hartl DL and Clark AG (2007) Principles of Population Genetics. Sunderland, MA: Sinauer Associates.

25. Storey J (2002) A Direct Approach to False Discovery Rates. J R Statist Soc B 64: 479-498.

26. Tyagi S, Gupta P, Saini AS, Kaushal C, Sharma S (2011) The peroxisome proliferator-activated receptor: A family of nuclear receptors role in various diseases. J Adv Pharm Technol Res 2: 236-240.

27. Altshuler D, Hirschhorn JN, Klannemark M, Lindgren CM, Vohl MC, et al. (2000) The common PPARgamma Pro12Ala polymorphism is associated with decreased risk of type 2 diabetes. Nat Genet 26: 76-80.

28. Saxena R, Voight BF, Lyssenko V, Burtt NP, de Bakker PI, et al. (2007) Genome-wide association analysis identifies loci for type 2 diabetes and triglyceride levels. Science 316: 1331-1336.

29. Zeggini E, Weedon MN, Lindgren CM, Frayling TM, Elliott KS, et al. (2007) Replication of genome-wide association signals in UK samples reveals risk loci for type 2 diabetes. Science 316: 1336-1341.

30. Chistiakov DA, Potapov VA, Khodirev DS, Shamkhalova MS, Shestakova MV, et al. (2010) The PPARgamma Pro12Ala variant is associated with insulin sensitivity in Russian normoglycaemic and type 2 diabetic subjects. Diab Vasc Dis Res 7: 56-62.

31. Jaziri R, Lobbens S, Aubert R, Pean F, Lahmidi S, et al. (2006) The PPARG Pro12Ala polymorphism is associated with a decreased risk of developing hyperglycemia over 6 years and combines with the effect of the APM1 G-11391A single nucleotide polymorphism: the Data From an Epidemiological Study on the Insulin Resistance Syndrome (DESIR) study. Diabetes 55: 1157-1162.

32. Meirhaeghe A, Boreham CA, Murray LJ, Richard F, Davey SG, et al. (2007) A possible role for the PPARG Pro12Ala polymorphism in preterm birth. Diabetes 56: 494-498.

33. Gong Z, Xie D, Deng Z, Bostick RM, Muga SJ, et al. (2005) The PPAR{gamma} Pro12Ala polymorphism and risk for incident sporadic colorectal adenomas. Carcinogenesis 26: 579-585.

34. Watson AD, Berliner JA, Hama SY, La Du BN, Faull KF, et al. (1995) Protective effect of high density lipoprotein associated paraoxonase. Inhibition of the biological activity of minimally oxidized low density lipoprotein. J Clin Invest 96: 2882-2891.

35. Sanghera DK, Aston CE, Saha N, Kamboh MI (1998) DNA polymorphisms in two paraoxonase genes (PON1 and PON2) are associated with the risk of coronary heart disease. Am J Hum Genet 62: 36-44.

36. Hegele RA, Connelly PW, Scherer SW, Hanley AJ, Harris SB, et al. (1997) Paraoxonase-2 gene (PON2) G148 variant associated with elevated fasting plasma glucose in noninsulin-dependent diabetes mellitus. J Clin Endocrinol Metab 82: 3373-3377.

37. Martinelli N, Girelli D, Olivieri O, Stranieri C, Trabetti E, et al. (2004) Interaction between smoking and PON2 Ser311Cys polymorphism as a determinant of the risk of myocardial infarction. Eur J Clin Invest 34: 14-20.

38. Wang Y, Luk AO, Ma RC, So WY, Tam CH, et al. (2010) Independent predictive roles of eotaxin Ala23Thr, paraoxonase 2 Ser311Cys and beta-adrenergic receptor Trp64Arg polymorphisms on cardiac disease in Type 2 Diabetes--an 8-year prospective cohort analysis of 1297 patients. Diabet Med 27: 376-383.

39. Weber WW, Hein DW (1985) N-acetylation pharmacogenetics. Pharmacol Rev 37: 25-79.

40. Vatsis KP, Martell KJ, Weber WW (1991) Diverse point mutations in the human gene for polymorphic N-acetyltransferase. Proc Natl Acad Sci U S A 88: 6333-6337.

41. Li YN, Gulati S, Baker PJ, Brody LC, Banerjee R, et al. (1996) Cloning, mapping and RNA analysis of the human methionine synthase gene. Hum Mol Genet 5: 1851-1858.

42. Christensen B, Arbour L, Tran P, Leclerc D, Sabbaghian N, et al. (1999) Genetic polymorphisms in methylenetetrahydrofolate reductase and methionine synthase, folate levels in red blood cells, and risk of neural tube defects. Am J Med Genet 84: 151-157.

43. Masud R, Qureshi IZ (2011) Tetra primer ARMS-PCR relates folate/homocysteine pathway genes and ACE gene polymorphism with coronary artery disease. Mol Cell Biochem 355: 289-297.

44. Jaiswal AK, McBride OW, Adesnik M, Nebert DW (1988) Human dioxin-inducible cytosolic NAD(P)H:menadione oxidoreductase. cDNA sequence and localization of gene to chromosome 16. J Biol Chem 263: 13572-13578.

45. Guha N, Chang JS, Chokkalingam AP, Wiemels JL, Smith MT, et al. (2008) NQO1 polymorphisms and de novo childhood leukemia: a HuGE review and meta-analysis. Am J Epidemiol 168: 1221-1232.

46. Kolesar JM, Dahlberg SE, Marsh S, McLeod HL, Johnson DH, et al. (2011) The NQO1*2/*2 polymorphism is associated with poor overall survival in patients following resection of stages II and IIIa non-small cell lung cancer. Oncol Rep 25: 1765-1772.

47. Pandith AA, Khan NP, Shah ZA, Shah AM, Wani SM, et al. (2011) Association of bladder cancer risk with an NAD(P)H:quinone oxidoreductase polymorphism in an ethnic Kashmiri population. Biochem Genet 49: 417-426.

48. Ding R, Lin S, Chen D (2012) Association of NQO1 rs1800566 polymorphism and the risk of colorectal cancer: a meta-analysis. Int J Colorectal Dis .

49. Rothman N, Smith MT, Hayes RB, Traver RD, Hoener B, et al. (1997) Benzene poisoning, a risk factor for hematological malignancy, is associated with the NQO1 609C-->T mutation and rapid fractional excretion of chlorzoxazone. Cancer Res 57: 2839-2842.

50. Fagerholm R, Hofstetter B, Tommiska J, Aaltonen K, Vrtel R, et al. (2008) NAD(P)H:quinone oxidoreductase 1 NQO1*2 genotype (P187S) is a strong prognostic and predictive factor in breast cancer. Nat Genet 40: 844-853.

51. Ehret GB, Munroe PB, Rice KM, Bochud M, Johnson AD, et al. (2011) Genetic variants in novel pathways influence blood pressure and cardiovascular disease risk. Nature 478: 103-109.

52. Takabatake N, Toriyama S, Igarashi A, Tokairin Y, Takeishi Y, et al. (2009) A novel polymorphism in CDC6 is associated with the decline in lung function of ex-smokers in COPD. Biochem Biophys Res Commun 381: 554-559.

53. Rouleau M, Patel A, Hendzel MJ, Kaufmann SH, Poirier GG (2010) PARP inhibition: PARP1 and beyond. Nat Rev Cancer 10: 293-301.

54. Yu H, Ma H, Yin M, Wei Q (2011) Association between PARP-1 V762A polymorphism and cancer susceptibility: a meta-analysis. Genet Epidemiol .

55. Kuczewski N, Porcher C, Lessmann V, Medina I, Gaiarsa JL (2009) Activity-dependent dendritic release of BDNF and biological consequences. Mol Neurobiol 39: 37-49.

56. Chen ZY, Jing D, Bath KG, Ieraci A, Khan T, et al. (2006) Genetic variant BDNF (Val66Met) polymorphism alters anxiety-related behavior. Science 314: 140-143.

57. Rybakowski JK (2008) BDNF gene: functional Val66Met polymorphism in mood disorders and schizophrenia. Pharmacogenomics 9: 1589-1593.

58. Savage DB, Zhai L, Ravikumar B, Choi CS, Snaar JE, et al. (2008) A prevalent variant in PPP1R3A impairs glycogen synthesis and reduces muscle glycogen content in humans and mice. PLoS Med 5: e27.

59. Mammarella S, Creati B, Staniscia T, Verginelli F, Manzoli L, et al. (2007) Obesity modifies the effects of the Asp905Tyr variant of PPP1R3A on risk of type 2 diabetes and insulin sensitivity. Diabetes Obes Metab 9: 759-761.

60. Vogel BE, Hedgecock EM (2001) Hemicentin, a conserved extracellular member of the immunoglobulin superfamily, organizes epithelial and other cell attachments into oriented line-shaped junctions. Development 128: 883-894.

61. Fisher SA, Rivera A, Fritsche LG, Keilhauer CN, Lichtner P, et al. (2007) Case-control genetic association study of fibulin-6 (FBLN6 or HMCN1) variants in age-related macular degeneration (AMD). Hum Mutat 28: 406-413.

62. Bojanowski CM, Tuo J, Chew EY, Csaky KG, Chan CC (2005) Analysis of Hemicentin-1, hOgg1, and E-selectin single nucleotide polymorphisms in age-related macular degeneration. Trans Am Ophthalmol Soc 103: 37-44.

63. Fuse N, Miyazawa A, Mengkegale M, Yoshida M, Wakusawa R, et al. (2006) Polymorphisms in Complement Factor H and Hemicentin-1 genes in a Japanese population with dry-type age-related macular degeneration. Am J Ophthalmol 142: 1074-1076.

64. Slaugenhaupt SA, Blumenfeld A, Gill SP, Leyne M, Mull J, et al. (2001) Tissue-specific expression of a splicing mutation in the IKBKAP gene causes familial dysautonomia. Am J Hum Genet 68: 598-605.

65. Anderson SL, Coli R, Daly IW, Kichula EA, Rork MJ, et al. (2001) Familial dysautonomia is caused by mutations of the IKAP gene. Am J Hum Genet 68: 753-758.

66. Chow RL, Volgyi B, Szilard RK, Ng D, McKerlie C, et al. (2004) Control of late off-center cone bipolar cell differentiation and visual signaling by the homeobox gene Vsx1. Proc Natl Acad Sci U S A 101: 1754-1759.

67. Heon E, Greenberg A, Kopp KK, Rootman D, Vincent AL, et al. (2002) VSX1: a gene for posterior polymorphous dystrophy and keratoconus. Hum Mol Genet 11: 1029-1036.

68. Ruiz-Perez VL, Ide SE, Strom TM, Lorenz B, Wilson D, et al. (2000) Mutations in a new gene in Ellis-van Creveld syndrome and Weyers acrodental dysostosis. Nat Genet 24: 283-286.

69. Lasa A, Piccolo F, de DC, Jeanpierre M, Colomer J, et al. (1998) Severe limb girdle muscular dystrophy in Spanish gypsies: further evidence for a founder mutation in the gamma-sarcoglycan gene. Eur J Hum Genet 6: 396-399.

70. Engert JC, Dore C, Mercier J, Ge B, Betard C, et al. (1999) Autosomal recessive spastic ataxia of Charlevoix-Saguenay (ARSACS): high-resolution physical and transcript map of the candidate region in chromosome region 13q11. Genomics 62: 156-164.

71. Arita K, South AP, Hans-Filho G, Sakuma TH, Lai-Cheong J, et al. (2008) Oncostatin M receptor-beta mutations underlie familial primary localized cutaneous amyloidosis. Am J Hum Genet 82: 73-80.

72. Verhoeven K, De JP, Van de PT, Nelis E, Zwijsen A, et al. (2003) Slowed conduction and thin myelination of peripheral nerves associated with mutant rho Guanine-nucleotide exchange factor 10. Am J Hum Genet 73: 926-932.

73. Ptacek LJ, Tawil R, Griggs RC, Engel AG, Layzer RB, et al. (1994) Dihydropyridine receptor mutations cause hypokalemic periodic paralysis. Cell 77: 863-868.

74. Stewart SL, Hogan K, Rosenberg H, Fletcher JE (2001) Identification of the Arg1086His mutation in the alpha subunit of the voltage-dependent calcium channel (CACNA1S) in a North American family with malignant hyperthermia. Clin Genet 59: 178-184.

75. Yang P, Diener DR, Yang C, Kohno T, Pazour GJ, et al. (2006) Radial spoke proteins of Chlamydomonas flagella. J Cell Sci 119: 1165-1174.

76. Castleman VH, Romio L, Chodhari R, Hirst RA, de Castro SC, et al. (2009) Mutations in radial spoke head protein genes RSPH9 and RSPH4A cause primary ciliary dyskinesia with central-microtubular-pair abnormalities. Am J Hum Genet 84: 197-209.

77. Reddi AH, Reddi A (2009) Bone morphogenetic proteins (BMPs): from morphogens to metabologens. Cytokine Growth Factor Rev 20: 341-342.

78. Babu RL, Wilson SG, Dick IM, Islam FM, Devine A, et al. (2005) Bone mass effects of a BMP4 gene polymorphism in postmenopausal women. Bone 36: 555-561.

79. Thye T, Vannberg FO, Wong SH, Owusu-Dabo E, Osei I, et al. (2010) Genome-wide association analyses identifies a susceptibility locus for tuberculosis on chromosome 18q11.2. Nat Genet 42: 739-741.

80. al-Marri MR (2001) Childhood tuberculosis in the State of Qatar: the effect of a limited expatriate screening programme on the incidence of tuberculosis. Int J Tuberc Lung Dis 5: 831-837.

81. Alsoub H, Al Alousi FS (2001) Miliary tuberculosis in Qatar: a review of 32 adult cases. Ann Saudi Med 21: 16-20.

82. Al-Khal AL, Bener A, Enarson DA (2005) Tuberculosis among garment workers in an Arabian developing country: State of Qatar. Arch Environ Occup Health 60: 295-298.

83. Bener, A. Annual Health Report. Hamad Medical Corporation, Doha, Qatar. 2010. http://site.hmc.org.qa/msrc/AHR2010/Pdf/Editorialboard.pdf [last accessed 7/13/12]

84. Carr DW, Stofko-Hahn RE, Fraser ID, Bishop SM, Acott TS, et al. (1991) Interaction of the regulatory subunit (RII) of cAMP-dependent protein kinase with RII-anchoring proteins occurs through an amphipathic helix binding motif. J Biol Chem 266: 14188-14192.

85. Bener A, Ayub H, Kakil R, Ibrahim W (2008) Patterns of cancer incidence among the population of Qatar: a worldwide comparative study. Asian Pac J Cancer Prev 9: 19-24.

86. Dolphin CT, Beckett DJ, Janmohamed A, Cullingford TE, Smith RL, et al. (1998) The flavin-containing monooxygenase 2 gene (FMO2) of humans, but not of other primates, encodes a truncated, nonfunctional protein. J Biol Chem 273: 30599-30607.

87. Whetstine JR, Yueh MF, McCarver DG, Williams DE, Park CS, et al. (2000) Ethnic differences in human flavin-containing monooxygenase 2 (FMO2) polymorphisms: detection of expressed protein in African-Americans. Toxicol Appl Pharmacol 168: 216-224.

88. Krueger SK, Siddens LK, Martin SR, Yu Z, Pereira CB, et al. (2004) Differences in FMO2*1 allelic frequency between Hispanics of Puerto Rican and Mexican descent. Drug Metab Dispos 32: 1337-1340.

89. Waness A, El-Sameed YA, Mahboub B, Noshi M, Al-Jahdali H, et al. (2011) Respiratory disorders in the Middle East: a review. Respirology 16: 755-766.

90. Wadvalla BA (2011) Pulmonary diseases may be highest in the Middle East. Nature Middle East.

91. Saccone SF, Saccone NL, Swan GE, Madden PA, Goate AM, et al. (2008) Systematic biological prioritization after a genome-wide association study: an application to nicotine dependence. Bioinformatics 24: 1805-1811.

92. Turner N, Mason PJ, Brown R, Fox M, Povey S, et al. (1992) Molecular cloning of the human Goodpasture antigen demonstrates it to be the alpha 3 chain of type IV collagen. J Clin Invest 89: 592-601.

93. Longo I, Porcedda P, Mari F, Giachino D, Meloni I, et al. (2002) COL4A3/COL4A4 mutations: from familial hematuria to autosomal-dominant or recessive Alport syndrome. Kidney Int 61: 1947-1956.

94. Stabuc-Silih M, Ravnik-Glavac M, Glavac D, Hawlina M, Strazisar M (2009) Polymorphisms in COL4A3 and COL4A4 genes associated with keratoconus. Mol Vis 15: 2848-2860.

95. Wu XJ, Qin L, Zhou JY, Cheng JL, Shen MY, et al. (2007) [Relationship between polymorphism of urotensin II gene and type 2 diabetes in pedigrees]. Zhonghua Yi Xue Yi Chuan Xue Za Zhi 24: 656-659.

96. Tan YJ, Fan ZT, Yang HX (2006) [Role of urotensin II gene in the genetic susceptibility to gestational diabetes mellitus in northern Chinese women]. Zhonghua Fu Chan Ke Za Zhi 41: 732-735.

97. Sun HX, Du WN, Zuo J, Wu GD, Shi GB, et al. (2002) [The association of two single nucleotide polymorphisms in PRKCZ and UTS2 respectively with type 2 diabetes in Han people of northern China]. Zhongguo Yi Xue Ke Xue Yuan Xue Bao 24: 223-227.

98. Zhu F, Ji L, Luo B (2002) [The role of urotensin II gene in the genetic susceptibility to type 2 diabetes in Chinese population]. Zhonghua Yi Xue Za Zhi 82: 1473-1475.

99. Saez ME, Smani T, Ramirez-Lorca R, Diaz I, Serrano-Rios M, et al. (2011) Association analysis of urotensin II gene (UTS2) and flanking regions with biochemical parameters related to insulin resistance. PLoS One 6: e19327.

100. Suzuki S, Wenyi Z, Hirai M, Hinokio Y, Suzuki C, et al. (2004) Genetic variations at urotensin II and urotensin II receptor genes and risk of type 2 diabetes mellitus in Japanese. Peptides 25: 1803-1808.

101. Song BL, Wang CH, Yao XM, Yang L, Zhang WJ, et al. (2006) Human acyl-CoA:cholesterol acyltransferase 2 gene expression in intestinal Caco-2 cells and in hepatocellular carcinoma. Biochem J 394: 617-626.

102. He X, Lu Y, Saha N, Yang H, Heng CK (2005) Acyl-CoA: cholesterol acyltransferase-2 gene polymorphisms and their association with plasma lipids and coronary artery disease risks. Hum Genet 118: 393-403.

103. Teslovich TM, Musunuru K, Smith AV, Edmondson AC, Stylianou IM, et al. (2010) Biological, clinical and population relevance of 95 loci for blood lipids. Nature 466: 707-713.

104. Fabre A, Martinez-Vinson C, Roquelaure B, Missirian C, Andre N, et al. (2011) Novel mutations in TTC37 associated with tricho-hepato-enteric syndrome. Hum Mutat 32: 277-281.

105. Cavanillas ML, Fernandez O, Comabella M, Alcina A, Fedetz M, et al. (2011) Replication of top markers of a genome-wide association study in multiple sclerosis in Spain. Genes Immun 12: 110-115.

106. McKenna A, Hanna M, Banks E, Sivachenko A, Cibulskis K, et al. (2010) The Genome Analysis Toolkit: a MapReduce framework for analyzing next-generation DNA sequencing data. Genome Res 20: 1297-1303.
