# Supplementary material for: Exome Sequencing of Only Seven Qataris Identifies Potentially Deleterious Variants in the Qatari Population
Source: PLoS One. 2012 Nov 6;7(11):e47614. doi: 10.1371/journal.pone.0047614 (PMC3490971; doi:10.1371/journal.pone.0047614)
Supplement: Web Resource S1 — SNPs genotyped in seven Qatari exomes. (PDF) [file pone.0047614.s010.pdf]

Web Resource S1. SNPs genotyped in seven Qatari exomes. Shown is all data included in the analysis for 126,924 autosomal SNPs genotyped in seven Qatari exomes (QE7) in autosomal chromosome (1-22). SNP information includes: position on human reference genome assembly GRCh37, NCBI dbSNP134 rsID, reference and alternate allele verified to be same in QE7 and 1000 Genomes. 1000 Genomes Project continental allele frequency includes alternate allele frequency in October 2011Integrated Phase 1 Variant Set Release of genotypes for n=1,092 individuals from four continents, sequenced by the 1000 Genomes Project (Table S3). QE7 allele count information includes: allele count in 7 Qatari exomes (QE7), maximum 14, followed by allele count for Q1_1, Q1_2, Q1_3, Q2_1, Q2_2, Q3_1, Q3_2 individuals. Functional classification information includes gene symbol from Consensus Coding Sequence (CCDS) NCBI database [8], coding function (noncoding, silent, missense, splice, nonsense); amino acid substitution and prediction of deleterious for missense SNPs from SIFT [11] or PolyPhen2 [12]; health-linked phenotype information from OMIM [15], HGMD [16], PharmGKB [17] or HUGE [18] database. Assessment of enriched allele frequency results include Fst and FDR when comparing QE7 and continental allele frequency; classification as enriched allele frequency *vs* each continent and *vs* at least one continent, the number of continents where the allele frequency is significantly different, significantly higher, and significantly lower. Last column lists Affymetrix 5.0 probeset matching the dbSNP rsID (see http://mezeylab.cb.bscb.cornell.edu/ResearchEvolGeno.aspx).
